# How to Characterize Covalent Adaptable Networks: A User Guide

**DOI:** 10.1021/acspolymersau.5c00004

**Published:** 2025-04-17

**Authors:** Dimitri Berne, Sidonie Laviéville, Eric Leclerc, Sylvain Caillol, Vincent Ladmiral, Camille Bakkali-Hassani

**Affiliations:** 119128 ICGM, Univ Montpellier, CNRS, ENSCM, Montpellier, France, www.enscm.fr

**Keywords:** Covalent Adaptable Network, synthetic strategies, molecular model reactions, cross-linking monitoring, swelling tests, thermo-mechanical properties, linear rheology, suitable rheological models

## Abstract

Since the seminal works on thermoreversible covalent networks followed by the discovery of vitrimers by L. Leibler and co-workers in 2011, numerous chemistries and strategies have flourished to design covalent adaptable networks (CANs) thus opening a novel research field. Using reversible covalent bonds that have been known for decades in molecular chemistry, CANs combine both the rheological characteristics of thermosets (chemically cross-linked networks, insoluble and infusible) and those of thermoplastics (entangled polymer chains able to be dissolved and to flow above their glass transition temperature). The aim of this tutorial review is to provide polymer chemists with guidelines to precisely and properly characterize CANs. Depending on the nature of the exchange mechanism (dissociative, associative, or a combination of both), on the kinetics of exchange, and on the cross-link density, characteristic relaxation times can vary from less than a second to a few hours. The time scale and distribution of relaxation times influence the rheological experiments and models that should be used. The present didactic review provides, from the rich recent literature, a guideline for adequate material characterizations and rheological measurements (and theoretical models applicable) that have been used to study CAN viscoelastic and thermomechanical properties.

## Graphical abstract

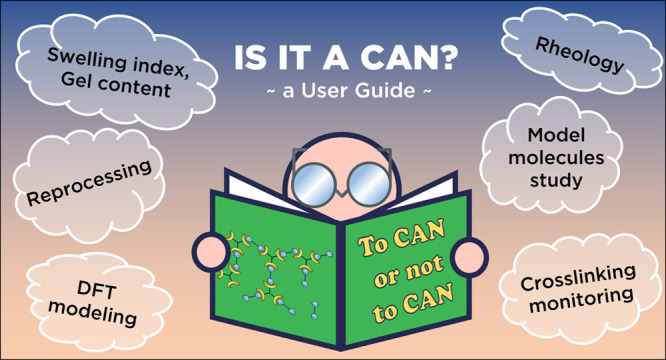

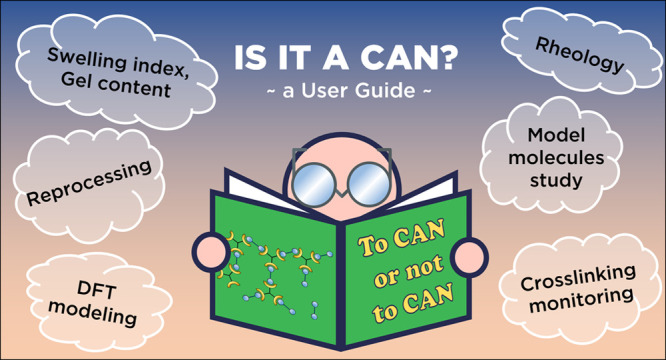

## Introduction

Conceptual advances in polymer chemistry often take advantage of a deeper understanding of organic chemistry reactivity at the molecular level.^1^ A typical example is the development of reversible deactivation radical polymerization methodologies in the 1990s which was derived from advances in radical chemistry.^2^ The development of dynamic covalent chemistry and its application in material science to covalent adaptable networks (CANs) and to self-healing polymers itself derive from tools and concepts coming from Constitutional Dynamic Chemistry (CDC, also known as DCC: Dynamic Combinatorial Chemistry).^3^ CDC emerged in the 1990s with the pioneering works of Sanders and co-workers as a strategy to generate molecular libraries by reaching thermodynamic equilibrium through reversible covalent and noncovalent reactions. This approach has not only provided chemists with a powerful tool to identify thermodynamic minima but has also led to the selective preparation of complex molecular architectures. Dynamic covalent reactions employed in CDC include, among others, acyl exchange (e.g., transesterification, transamidation, transcarbamoylation), Diels–Alder reactions, alkene metathesis, oxime exchanges, or disulfide and acetal exchange, while hydrogen bonding and metal ligand-exchange have been used as noncovalent interactions.^4,5^ Later on, these reactions and their dynamic nature (with or without activation) have been introduced in polymer chemistry to combine, in a new class of materials, properties which were considered thus far as antagonistic. The so-called CANs were designed to merge the properties of thermosets and thermoplastics and provide smart, stimuli-responsive, and sustainable materials for applications in various fields such as biomedicine, energy storage and conversion, transportation, and construction, for example. The principle was to introduce covalent exchanges in a chemically cross-linked network to retain the dimensional stability of thermosets and the ability of thermoplastics to be reshaped upon heating.

Alternatively to CANs, self-healing materials generally involve noncovalent bonds and are especially interesting for applications that do not require the use of high temperatures, as for instance in the biomedical field^6^ or in soft robotics.^7^ Self-healing polymers based solely on noncovalent labile bonds suffer from the fact that the interactions allowing mechanical recovery become weak or even insignificant above a certain temperature; their thermal behavior then approaches that of usual thermoplastics, leading to an uncontrolled loss of mechanical properties. This review focuses on dynamic networks based on covalent bonds (CANs).

## Synthetic Strategies and Classification of CANs

Two main strategies have been developed to design CANs (Figure 1).^8^ They can be obtained through a one-step process from multifunctional monomer mixtures (average functionality, *f̅* > 2). In this case, the dynamic bonds are either already present by appropriate monomers design^9–11^ or result from the chain growth/cross-linking reaction.^12–16^ Alternatively, macromolecules (thermoplastics) can be cross-linked either by a mechanism which creates reversible covalent bonds or by employing a cross-linker which already contains such dynamic moieties.^17^ This approach called “vitrimerization” sometimes requires prior modification of the polymer backbone to introduce reactive pendant groups.^18,19^ Polyethylene,^18,20–22^ polybutylene,^23^ polysiloxanes,^24^ polyalkyl (meth)­acrylates,^22,25,26^ and polystyrene^27,28^ cross-linked with functional groups able to undergo exchange reactions have been employed to generate so-called “vitrimerized thermoplastics”. Finally, starting from thermosets containing potentially exchangeable bonds (esters, urethanes, etc.), the *a posteriori* incorporation of catalysts can confer dynamic properties to the material, thus offering an interesting pathway to reshape/recycle thermoset wastes.^29^

**Figure 1:**
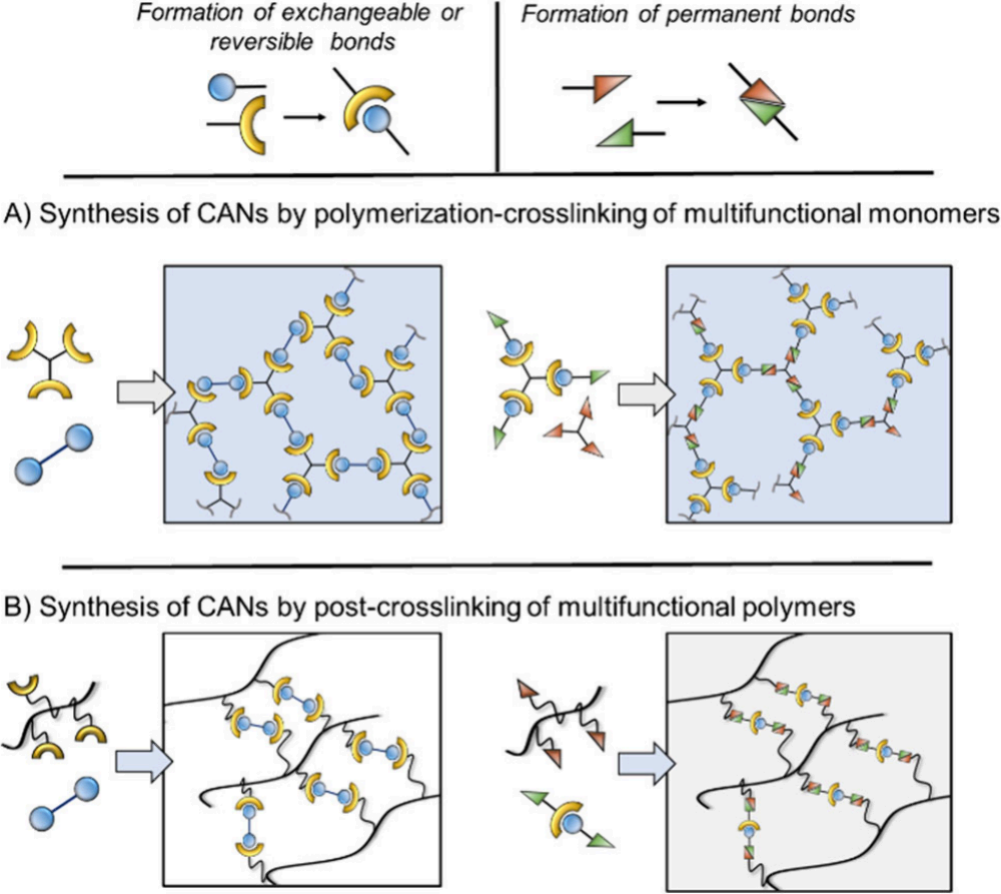
Synthetic strategies for the preparation of CANs A) by polymerization/cross-linking of multifunctional monomers and B) by cross-linking of functional polymer backbone.

Covalent exchange reactions and related materials (Figure 2) have been reviewed in several articles^30–33^ and are usually classified into two categories according to the type of exchange mechanism: dissociative or associative CANs (also called transient networks, reversible networks or dynamic covalent polymer networks). Transesterification,^34–37^ transamination,^38,39^ Diels–Alder exchange,^40–43^ thia-Michael exchange,^44–46^ transcarbamoylation,^47–49^ and transimination^50–52^ are among the most commonly used exchange reactions. Although some of these reactions are effective in the absence of any promoter (transamination, transimination, Diels–Alder exchange), many require the use of catalysts to occur at temperatures below the degradation temperature of the polymer matrix. The dynamic character of CANs can be triggered via thermal or photochemical activation but also by mechanical stimulation in rarer cases.^16,53,54^ CANs were initially classified into two categories according to the nature of the mechanism involved in the exchange process: dissociative CANs, which are based on a cleavage/reformation equilibrium of the exchangeable bonds, and associative CANs, in which the exchange reaction proceeds through an associated transition state (a new bond is formed either before the cleavage of another bond or in a concerted manner). The so-called vitrimers are a subclass of associative CAN in which the covalent exchange is thermally trigerred. These two distinct mechanistic pathways at the molecular level translates, at the macroscopic scale, into differences in the network topological rearrangements, and therefore lead to different rheological behavior.^55^ In the case of dissociative CANs, the equilibrium between associated and dissociated states can be theoretically totally shifted from cross-linked chemical structure to the liquid state. In practice, the decrease in cross-link density networks is often only observed at high temperature, and the material could behave as an associative CAN over a wide range of temperatures (with only an apparent almost constant cross-link density). In the case of associative CANs or vitrimers, the exchange proceeds generally through an addition–elimination mechanism, thus maintaining a constant cross-link density regardless of the temperature (Figure 2). Associative covalent exchange can also occur through the formation of a cyclobutene intermediate (metathesis of alkenes) or via concerted nucleophilic substitution (S_N_2) in the case of transalkylation.

**Figure 2:**
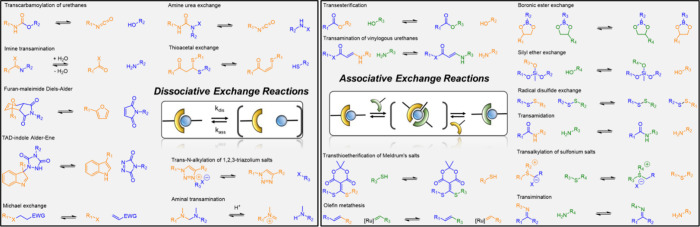
Typical covalent exchange reactions used in the Covalent Adaptable Network design.

Numerous articles and reviews summarizing the exchange reactions, strategies, properties, and potential applications of CANs in polymer science have been published. This tutorial review aims to provide a vade mecum to the neophytes in the field who would like to start working on dynamic networks based on covalent bonds (CANs). The methodology described here should help readers understand the rich scientific literature dedicated to CANs, and it should help them in making decisions during their own investigations. The intention is also to standardize the way of analyzing CANs thus allowing clearer identification and comparison of structure-properties relations. After a brief description of the general properties of thermoplastics and thermosets, characterization methods and models used to describe the rheological behavior of CANs are presented. Original systems (combining multiple exchange mechanisms) and emerging characterization methods of CANs are also discussed.

## Generalities and Methodology of CAN Characterization

Polymer materials are generally classified in two main families depending on their thermo-mechanical behavior and chemical landscapes (Figure 3).^57,58^ Thermoplastics are composed of linear (but also branched, dendritic, cyclic) macromolecules interacting through noncovalent intermolecular forces (hydrogen bonds, van der Waals, π-π stacking, etc.). Material cohesion is due to short-range interactions, chain entanglement, and long-range order (crystallinity) in the case of semicrystalline polymers. Thermoplastics are thus usually (re)­processable, recyclable, soluble in solvents, and capable of flowing at generally moderate temperature (above their melting or glass transition temperature). The second class of polymers consists of thermoset materials. Thermosets are chemically cross-linked networks, i.e. macromolecular chains linked to each other by permanent covalent bonds. They originate from the discovery of Bakelite at the beginning of the twentieth century.^59^ The chemically cross-linked structure affords outstanding properties, such as chemical resistance (they do not dissolve but only swell in good solvents), excellent mechanical properties, and thermal and dimensional stability. However, such properties are associated with a major drawback: they cannot be easily reprocessed or recycled. CANs are chemically cross-linked materials (thermosets) which contain reversible or exchangeable covalent bonds. From this definition, two levels of characterization should be considered. First, the study of CANs as thermosets is an essential prerequisite as it provides key data on these materials that are required for the following dynamic characterizations. Second, at higher temperature, the presence of exchangeable covalent bonds as well as the mechanism of exchange (associative or dissociative) has significant impact on the viscoelastic response of this class of material. Rheological analysis is the method of choice to establish the relationship between viscoelastic material behavior and molecular connectivity and dynamic. What follows are guidelines to readily and accurately characterize CAN properties. Prior to the description of rheological analyses and associated models, a set of required experiments is presented below.

**Figure 3:**
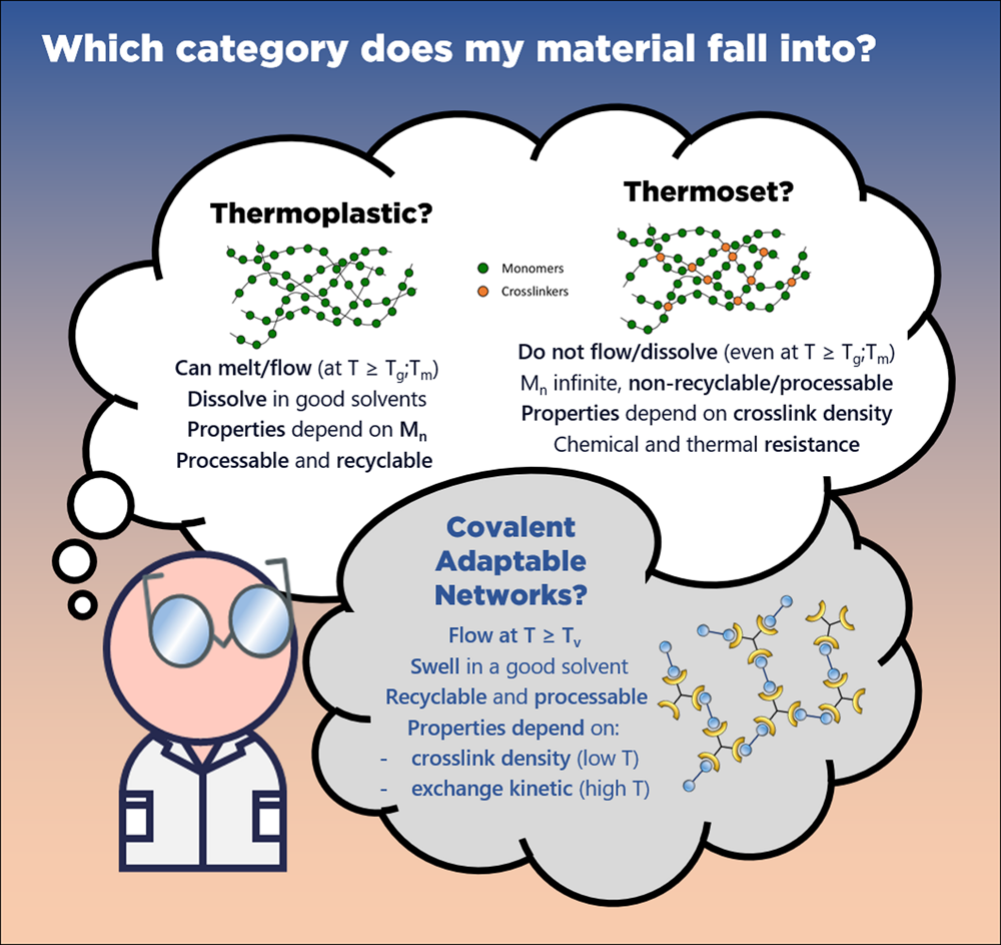
Main properties of thermosets, thermoplastics, and CANs.

Thermoplastics can be analyzed using conventional polymer characterization methods such as size exclusion chromatography, liquid NMR spectroscopy, or mass spectrometry, allowing the precise determination of molar mass, dispersity, and macromolecular structure. Those techniques require the dissolution of polymer in solvents and cannot be applied to thermosets, which are by definition insoluble and infusible. Thermoset’s characterization generally involves functional group analysis by FTIR or Raman spectroscopy, swelling experiments, and thermomechanical analysis (TGA, DSC, DMA, mechanical tests, etc.). For comprehensive reviews on thermosets characterizations, readers can refer to the review of Johnson et al.^60^ and the educational review by De Alwis Watuthanthridge et al.^61^ For an educational review specifically dedicated on linear rheology of vitrimers, readers can refer to the work of Ricarte et al.^62^ To characterize a CAN we proposed the following procedures highlighted in the blue box and illustrated in a comic strip in Figure 24.

1. **Molecular model reactions**. Small molecule studies allow investigation of the elementary chemical steps involved in both network synthesis and covalent exchanges. The influence of additives (catalysts), temperature, and other experimental conditions can be monitored at the molecular level. This monitoring gives access to the thermodynamic and kinetic parameters associated with the reaction leading to the network and with the exchange reactions and are generally performed using solution NMR spectroscopy and/or or chromatography techniques (i.e., GC/LC-MS). In addition, theoretical calculations (e.g., DFT calculations) could give additional information on the mechanism, plausible transition states and activation energy. Molecular reactions required to establish the relationship between molecular dynamics and material properties can be performed either before or after the following steps.
2. **Cross-linking Monitoring**. To follow the formation of a chemically cross-linked network, two main experiments can be performed. *In situ* FTIR (Fourier Transformed Infrared) spectroscopy allows one to monitor the evolution of chemical composition as a function of curing time and temperature. The polymerization/gelation kinetic, conversion, and occurrence of side reactions can be extracted from FTIR spectra. The gel time can be obtained through small amplitude oscillatory shear employing the multiwave mode of the rheometer to record simultaneously the storage (*G*′(ω)) and loss (*G*″(ω)) moduli at different frequencies. This experiment also provides information on the curing kinetic and stability of the formulation.
3. **Determination of gel content and swelling index**. The swelling test should be the first characterization to be performed after curing as it requires only solvent, glassware and a precision scale but allows one to discriminate rapidly between a thermoplastic and a thermoset while providing crucial insights on the nature of the material studied. Parameters such as the swelling index (solvent uptake), gel content and soluble fractions can be extracted/calculated directly from swelling tests. Additional analysis of the soluble fractions (solution NMR spectroscopy, mass spectrometry, etc..) should also be carried out to fully characterize the soluble fraction. Dissolution/depolymerization tests (with a reactive solvent) are also of interest to qualitatively demonstrate the occurrence of covalent exchange (and potential chemical recycling).
4. **Determination of the main transitions (*T***_**g**_ **,** ***T***_**m**_ **and** ***T*_**d**_) and suitable temperature window for rheology.** Thermogravimetric analysis (TGA) and differential scanning calorimetry (DSC) should be performed to determine respectively the onset of degradation (2 or 5 wt % loss) and the glass transition temperature. Additional information such as crystallinity, phase separation (e.g., if two *T*_g_ are observed etc.) can be also extracted from such measurements. Rheology experiments should be performed in the [*T*_g_ + 50 °C: *T*_d_ – 20 °C] range to avoid artifact from the glass transition (cooperative segmental relaxation) or from thermal degradation (chain scission, depolymerization, oxidation etc.).
5. **Mechanical tests and reprocessing experiments.** Mechanical tests (e.g., tensile, compression, bending) allow one to determine both the initial material properties (before reprocessing) and the potential range of application of the studied material. Young’s modulus, ultimate strength and strain at break can be extracted from stress–strain curves. Even if advanced rheological measurements are needed to determine the optimal conditions for reprocessing (time, temperature), initial reprocessing/reshaping tests should be performed to rapidly assess if the network is able to rearrange or not.
6. **Linear rheology (flow characterization).** Dynamic mechanical analysis (DMA) is used to characterize material properties over a wide range of temperature (from −150 °C and up to 600 °C depending on the apparatus). The existence of a rubbery plateau after *T*_g_ proves the thermoset structure while the rubbery modulus value is directly linked to the cross-link density. At high temperature, DMA can provide useful information to discriminate between an associative and dissociative network. Stress relaxation, creep-recovery and small amplitude oscillatory shear (SAOS) experiments allow one to determine the relaxation time (the ratio between viscosity and modulus) and its temperature dependency. Apart from the relaxation time, the accurate evolution of moduli (G_t_, G’_ω_, G”_ω_ or J_t_) or zero-shear viscosity (η_0_) with temperature measured by such methods sheds light on the exchange mechanism pathway (associative or dissociative) occurring in the chemical network.
7. **Reprocessability study.** The reprocessability study consists in grinding the CANs into powder prior to process them at a defined temperature either by compression molding or by other polymer processing techniques (e.g., extrusion, injection). The reprocessed materials should be extensively characterized by swelling tests, FTIR, DMA and tensile tests to evaluate the impact of the reprocessing cycle on cross-link density, chemical composition, mechanical properties and main thermal transitions (*T*_g_, *T*_m_ and *T*_d_). In addition, the impact of reprocessing cycle(s) on the covalent exchange efficiency (catalyst deactivation, side reactions etc.) should be evaluated by measuring/comparing the characteristic relaxation time before/after reprocessing.
8. **Establish the chemical/material properties relationship.** By combining the results obtained from molecular investigations (both experimental and theoretical), rheological characterizations and mechanical/reprocessing/recycling tests, it is possible to establish the relationship between the chemical exchange processes and the material thermomechanical (and flow/malleability) properties through the comparison of thermodynamic and kinetic parameters (activation energy, kinetic constants, etc.).

## Molecular Model Reactions and Modeling

The properties of CANs are deeply connected to the exchange reactions and their associated kinetics and mechanism, conferring them their dynamic behavior. Consequently, a close examination of these reactions at the molecular level is crucial to either accurately design the structure of the polymer matrix or to interpret the dynamic mechanical properties of the resulting network (even if discrepancies between molecular and macromolecular behavior can be sometimes observed.^63,64^ The following section describes the know-how needed to perform molecular investigations on exchange reactions. The study of molecular processes suggested here can be also used to determine the optimal conditions for the chemical network synthesis.^65^

### Qualitative Exchange Reactions

A standard prerequisite to the preparation of a CAN is to check the feasibility of the exchange reaction (if unprecedented) by performing small molecule studies (Figure 4). In the case of reactions which presumably involve a dissociative or metathesis-type mechanism, scrambling experiments between two judiciously designed substrates are often performed to check the rate of the reaction under appropriate thermal conditions. The design of vitrimers based on dioxaborolane exchanges was, for example, supported by a prior molecular study demonstrating the metathesis of two boronic esters bearing different diols and aromatic substituents in a few hours at 60 °C.^22^ Ideally, the thermodynamic bias of the scrambling reaction should not be marked, so that the four resulting species can be observed when equilibrium is reached.

**Figure 4:**
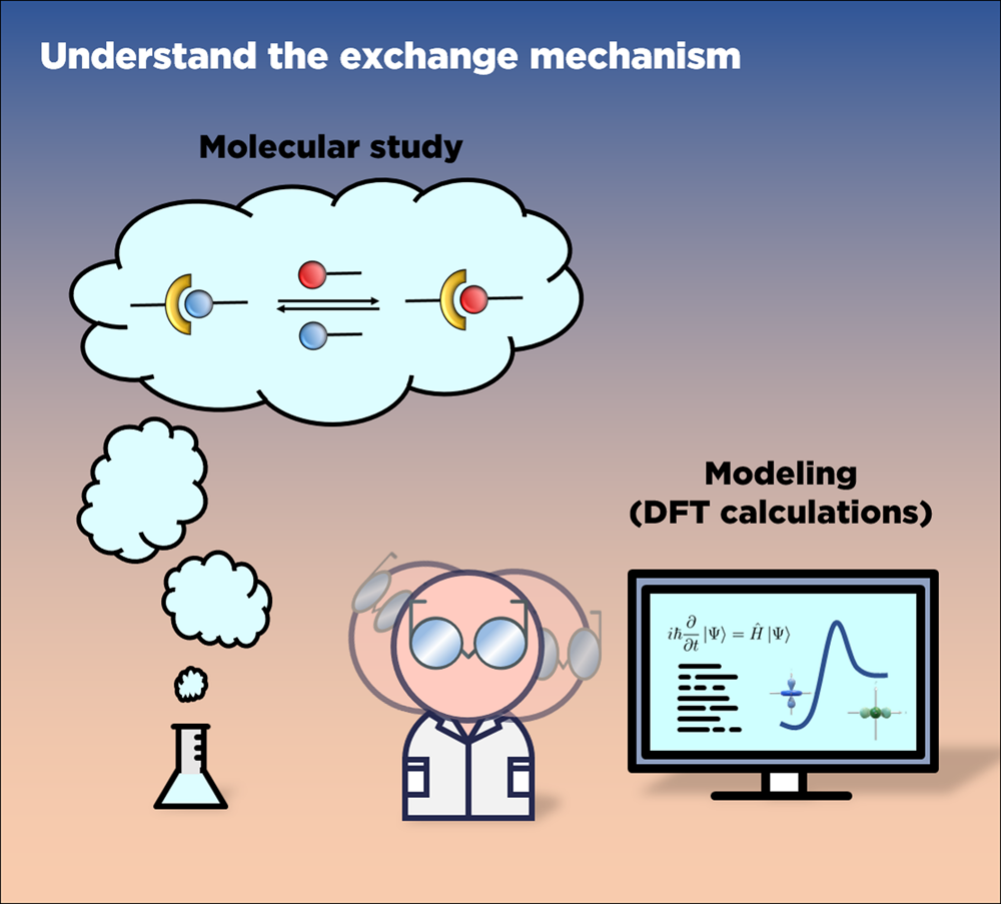
Characterization of the exchange mechanism by molecular studies and theoretical investigations.

However, competition-type experiments are more commonly used when the mechanism of the exchange reaction is presumably associative. In that case, only one exchangeable function is left to react with the hypothesized reactive partner, and the formation of the resulting function is monitored. Typically, transesterification or transamidation reactions are generally assessed by monitoring the formation of a new ester or amide from an ester or amide substrate and a competing amine or alcohol. The design of vinylogous urethane vitrimers was grounded on a model exchange reaction between an enaminoester and an excess of benzylamine resulting in the formation of a new enaminoester.^38^ For such reactions, an excess of the reactive partner (in that case, the amine) is often used to allow the faster formation of the exchange product and shift the equilibrium.

### Influence of Additives or Catalyst

A variety of catalysts have thus been used to improve and control the rate of exchange reactions within CANs, employing a catalyst decreases the activation energy of the exchange reactions thus allowing the exchange dynamics to speed up.^31^ Typical examples of acyl exchange catalysts include, among others, metal complexes such as tin compounds,^66–68^ zinc-based catalysts,^16,69,70^ titanium tetroxide derivatives,^71,72^ organocatalysts such as 1,5,7-triazabicyclodec-5-ene (TBD),^73,74^ 1,8-diazabicyclo[5.4.0]­undec-7-ene (DBU),^75^ phosphines,^75^ Brønsted acids (e.g., sulfonic acids)^76–78^ and even enzymes (Lipase TL).^65,79^ Along with catalytic activation, the so-called neighboring group participation (NGP) strategy has been developed to prepare catalyst-free CANs. The NGP effect relies on the introduction of a specific function in the proximity of the exchangeable groups in order to modify the rate of the exchange reaction or the exchange mechanism itself (e.g., intramolecular cyclization). Examples of NGP strategies are the formation of H-bonded transition states^16,77,80–85^ or of cyclic intermediates,^86–91^ and the introduction of activating groups (electron-withdrawing groups^92–101^ or chemical functions which act as catalysts such as tertiary amine^21,37,87,102–109^ ). The influence of additives or catalysts on such model reactions can also be examined in order to devise mechanistic hypotheses or discriminate possible pathways. The metathesis mechanism suggested for the direct exchange between two dioxaborolanes was for example challenged by using various amounts of methanol or water as an additive (Figure 5).^110^ This resulted in an acceleration of the exchange reactions via ring opening of the dioxaborolanes and subsequent diol exchanges between the two partners. This confirmed that such a mechanism, triggered by adventitious water (or another nucleophile), could be at work in the material rather than a direct metathesis.^111^ Similarly, the use of acidic or basic additives in the reaction of amines with vinylogous urethane allowed the authors to unveil the occurrence of two different mechanisms for this exchange reaction. Indeed, the addition of a catalytic amount of Bronsted acid increased the rate of the uncatalyzed reaction, but the measured activation energies of both reactions were close. In contrast, the addition of a catalytic amount of base slowed down the reaction and increased the activation energy.^78^ It was deduced from these results that the acid-catalyzed and uncatalyzed reactions on one hand and the reaction in the presence of base on the other hand proceeded through two different mechanisms. The first one was postulated to involve an iminium-type activation of the vinylogous urethane, while the second presumably proceeds through a direct aza-Michael addition of the competing amine. Interestingly, the occurrence of these two mechanisms in a vitrimer material was afterward evidenced by a dual temperature dependence observed in the stress relaxation experiments.^112^

**Figure 5:**
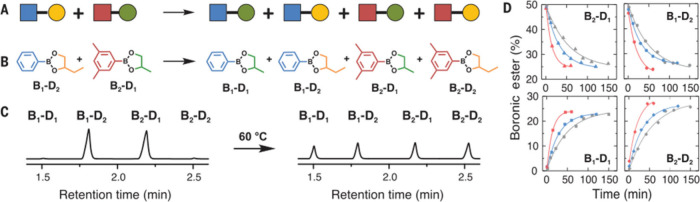
Scrambling experiments performed to characterize the exchange of dioxaborolane moieties A and B schematic of the molecular scrambling experiments between two dioxaborolane C and D GC-MS experiments and kinetic plots. From ref 22. Reprinted with permission from AAAS.

### Determination of Thermodynamic and/or Kinetic Parameters

The determination of key parameters in model exchange reactions can also be helpful in optimizing the design of the corresponding vitrimers. For example, it was anticipated that the use of bulky amines for the formation of urea bonds should allow the existence of an equilibrium between the product and the starting amine and isocyanate, which could prove to be useful for the study of exchange reactions and the design of urea-based CANs. This equilibrium was quickly evidenced by the authors using a small range of bulky secondary amines.^113^ However, the determination of the equilibrium and dissociation constants was crucial in choosing the best-fitted structure of the secondary amine for material purposes. Indeed, the equilibrium should be considerably displaced toward the urea function to ensure network formation and stability. Nevertheless, the dissociation reaction must remain sufficiently fast to allow for exchange reactions. This study allowed the authors to pick an adequate compromise and to design efficient recyclable poly­(urea-urethane) CANs.^114^

A thorough kinetic study of a model reaction can also be used to confirm the acceleration of an exchange reaction due to a postulated NGP-type activation. The rate enhancement of a transesterification reaction thanks to fluorine substitution has, for example, been evidenced by the experimental determination of transesterification rates for various nonfluorinated and fluorinated esters. A 47-fold acceleration has been observed for difluorinated esters compared to their nonfluorinated counterparts, while monofluorination only led to a marginal acceleration.^115^ This study grounded the design of catalyst-free transesterification vitrimers using such fluorinated esters.^101^

### DFT Calculations

DFT studies have also been used to challenge postulated mechanistic pathways, such as the two mechanisms supposedly at stake in dioxaborolane exchange, i.e. direct metathesis and nucleophile-assisted ring-opening. A recent study also modelized the different mechanistic pathways postulated for vinylogous urethane exchange and confirmed the plausibility of both iminium-type and aza-Michael mechanisms.^116^ The transesterification and transmidation rate acceleration induced by fluorine substitution, already observed experimentally, was also confirmed by DFT calculations.^115,117^ Finally, the promoting role of a tertiary amine in thia-Michael-type exchanges implemented in vitrimers was also unambiguously confirmed by the calculated Gibbs energy profile of the reaction.^118^

## Covalent Adaptable Network Characterizations

The design of a CAN involves the polymerization/gelation of multifunctional monomers (or macromonomers) as already discussed above. Prior to studying the network formation, it is important to determine the exact functionality (number of reactive functions per molecule) of each component of the system by chemical and/or NMR titration. Even for commercially available products, the exact concentration of reactive groups can differ from theoretical values due to the occurrence of side reactions (e.g., oligomerization, hydrolysis, etc.). For instance, oligomers are often detected in epoxy- or isocyanate-based formulations, thus leading to off-stoichiometric formulations. In addition, some chemical groups, e.g., oxiranes, are also able to homopolymerize generating permanent bonds and modifying both the static and dynamic properties of the resulting network.^119^

### Cross-Linking Monitoring

The gelation or cross-linking of a CAN (or thermoset) formulation is generally assessed by combining spectroscopic characterizations of chemical groups and gel time experiments. Among techniques, FTIR and especially the use of attenuated total reflection (ATR) mode allow capture, in a simple manner, of the chemical composition of a polymer network. The signature vibration of molecular dipole moment which is associated with a specific chemical function provides insight into the curing reaction nature, the conversion, and the occurrence of side reactions. Even if only a few micrometers are probed by the evanescent wave (penetration of the evanescent wave is given by eq 1), ATR-IR allows one to study glassy, elastomer and liquid samples if a strong contact between the cell and the sample is achieved. If the apparatus is equipped with a heating cell (Figure 6A), *in situ* monitoring of curing can be easily performed as depicted in Figure 6. For instance, an isothermal ATR-IR kinetic experiment (at 50 °C) of a CAN based on trifluoromethylated *N,S*-acetal^120^ is presented in Figure 6B. The cross-linking reaction was monitored thanks to the vibration associated with the stretching of C–F bonds which shift from 1320 cm^–1^ (imine monomer) to 1250 cm^–1^ during the formation of a *N,S-acetal* covalent adaptable network. The conversion at any time is given by eq 2. At the end of the curing, it is also advised that one record both the top and bottom IR signature of a material specimen to quickly evaluate if macroscopic phase separation has occurred during polymerization/gelation.

1 $$D_{p}=\frac{\lambda }{n_{1}2\pi \sqrt{\sin^{2}\theta -{\left(\frac{n_{2}}{n_{1}}\right)}^{2}}}$$

where n_1_ and n_2_ are the refractive indices of the ATR crystal and the sample, respectively, θ ≈ 45° is the incidence angle, and λ = 2.5 to 25 μm is the wavelength of mid-infrared radiation.

2 αImine=1−(A(t)®1320A0®1320)

where α_imine_ is the imine conversion; *A̅*_01320_ and ${\overset{®}{A_{\left(t\right)}\;}}_{1320}$ are the normalized absorbances of the imine groups before curing and after the reaction time *t*, respectively.

**Figure 6:**
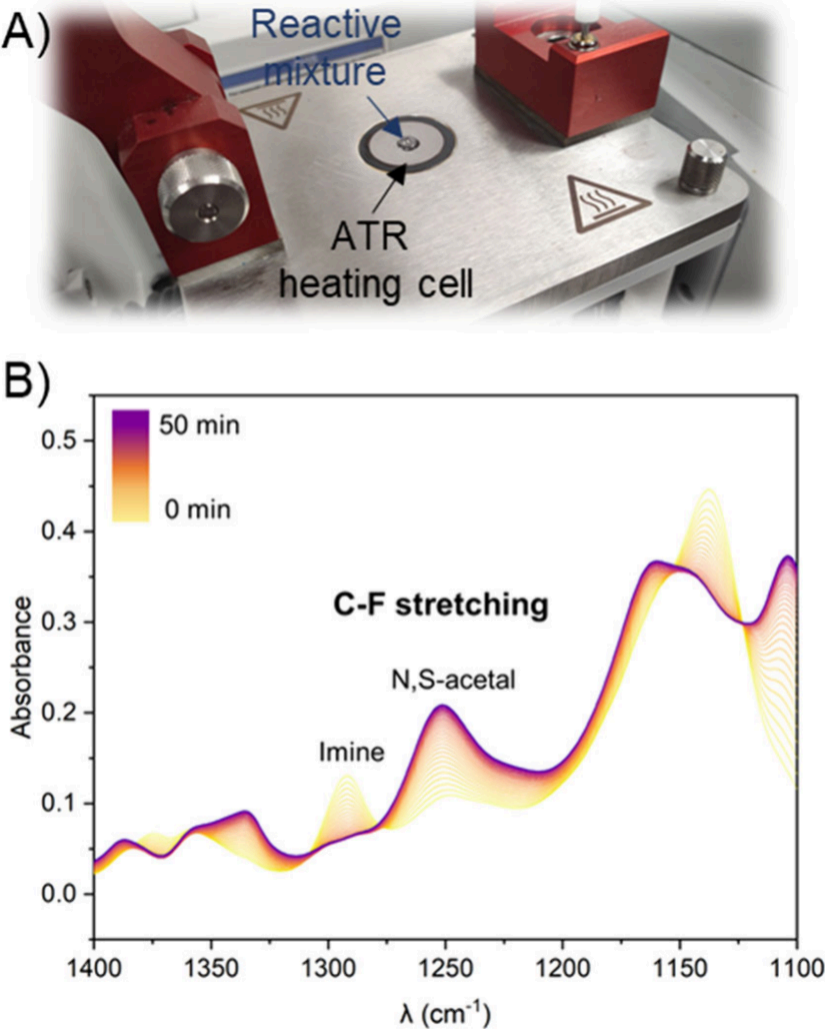
(A) ATR-IR apparatus equipped with a heating cell and (B) ATR-IR kinetic experiments. Reproduced from ref 120. Copyright 2024 American Chemical Society.

However, FTIR is limited to polar bonds, and the fingerprint region (below 1400 cm^–1^) can be complex to analyze. Raman spectroscopy is based on change in bond polarizability associated with the vibrational mode (inelastic diffusion of the photon). It is a complementary technique to FTIR (based on absorption by vibrational states), as active vibration modes in FTIR are not active in Raman and vice versa. In the field of CANs, Raman spectroscopy has cast light on CANs containing dynamic disulfide bonds (S–S),^121^ thiourethanes,^122^ or imines^123^ etc. which only weakly absorb infrared wavelength. In Raman scattering, incident photons interact with molecules bringing them to virtual energy states which can be problematic when the polymer network includes within its structure fluorophore or chromophores moieties. Both Raman and FTIR techniques can be coupled with other characterization methods such as microscopy^124^ and rheology^125^ providing valuable insight on phase separated materials^123^ or on transient network formed during mechanical perturbations.

In addition to spectroscopic characterizations, the gel time, which represents the instant at which the average molar mass diverges to infinity (the chemical gel), should be determined by Fourier transform mechanical spectroscopy. The evolution of storage G′_ω_ and loss shear modulus G″_ω_ as a function of reaction time in small amplitude oscillatory shear (SAOS) is depicted in Figure 7A for *N,S*-acetal network^120^ formation at 25 °C. Using SAOS and the multiwave mode of the rheometer to record simultaneously the storage (*G*′_ω_) and loss (*G*″_ω_) moduli at different frequencies^126^ enables the accurate determination of the liquid to gel transition (Figure 7B). The physical meaning of the different moduli is described below in the Rheology Basics section. According to the Winter–Chambon criterium, the relaxation modulus (G_*t*_) of the chemical network at the gel point follows a power law with G_*t*_ ∼ *t* ^–n^, where t is the time and n is the critical exponent^127^ (0 < *n* < 1). At the gel point, the loss factor tan­(δ_ω_) = *G*″_ω_/*G*′_ω_ is independent of ω. As shown in Figure 7B the gel time point is readily determined by the crossover point of the tan­(δ) plot at different frequencies. Violations of the Winter–Chambon criterion (i.e., nonintersecting of the loss tangents) can also occur as the model is only valid for stoichiometric balanced networks at temperatures sufficiently high above their glass transition temperature.^127^ As an example, Ilvasky et al. studied off-stoichiometric polyurethane and epoxide formulations where the gel point was solely reached when all the limiting reactants had been consumed.^128^ In this particular case, the loss tangents for both systems had a slight dependence on the frequency at the gel point. Apart from the exact determination of critical gel formation, the gel time experiment also provides insight on the curing kinetics, the influence of additives, and the thermal stability of the initial formulation (also called pot-life). For instance, Cramail and co-workers recently used SAOS experiments to highlight the influence of water during the formation of polyhydroxy-urethane chemical networks.^129^ They found out that small amounts of water (<5 wt %) shorten gel times (2- to 5-fold decrease) and suggested that water was able to enhance molecular mobility within PHU clusters and delayed vitrification.

**Figure 7:**
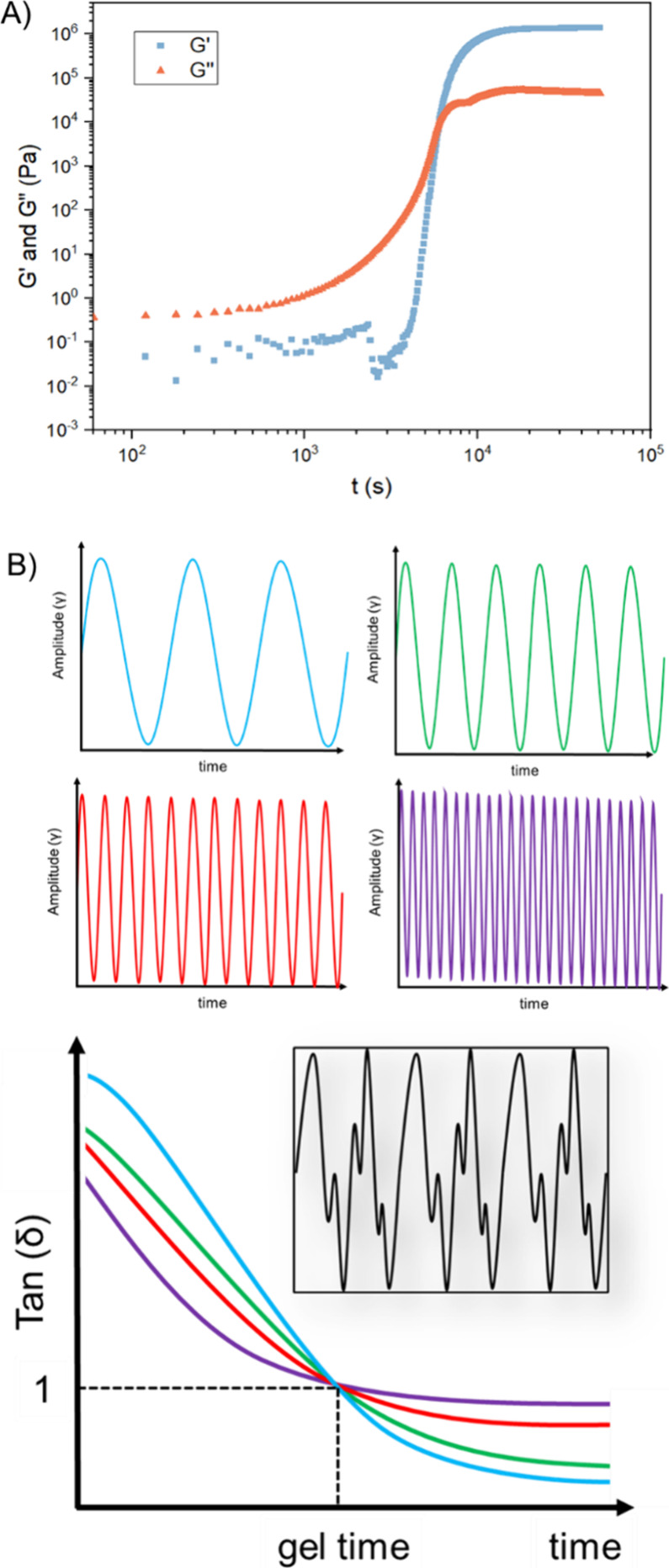
(A) Evolution of storage and loss modulus as a function of reaction time at 25 °C (ω = 1 rad·s^–1^, γ = 0.2%) for the curing of *N,S*-acetal CAN. Reproduced from ref 120. Copyright 2024 American Chemical Society. (B) Schematic representation of Fourier transform mechanical spectroscopy and evolution of loss factor tan­(δ) as a function of time at various frequencies.

Although structural characterization of cross-linked materials is very difficult, high resolution magic angle spinning NMR spectroscopy (HR-MAS NMR) was recently shown to be useful to monitor the chemical process at work during CAN formation.^120,130,131^ Even if it is more complex to set up compared to FTIR analysis and limited to materials capable to be sufficiently swollen by deuterated solvents, HR-MAS NMR spectroscopy is one of the rare (if not the only) techniques that can provide detailed insight on the chemical structure of CANs.

### Swelling/Solubility Tests

To assess the existence of a chemically cross-linked network, the swelling test is a rapid and simple experiment to perform. Apart from discriminating rapidly between a thermoset and thermoplastic, the presence of soluble polymers/oligomers trapped in the chemical networks can also modify the mechanical properties (plasticizing effect), i.e. the cross-link density, or dissipate mechanical energy.^132^ In addition, it can also impact the exchange dynamic of CANs by modifying the concentration of exchangeable groups. Hence, it is essential to first verify the insoluble character of these materials. A swelling test is performed by immersing (at least on triplicate) samples of a regular shape (m_0_) in an appropriate solvent. In the context of this review, an appropriate solvent is defined as one in which monomers or the non-cross-linked polymers are initially soluble and do not react with the exchangeable functions. During a swelling test, the polymer network is swollen by the solvent, and the network junctions and chains are pulled apart to accommodate the increasing volume fraction of solvent. These strained conformations result in a retractive force that tends to bring the network chains into more probable conformations. The equilibrium is reached when the entropy of dilution and the retractive network forces balance (m_1_ in Figure 8 corresponding to the mass of the swollen sample).^133^ In other words, for chemical networks with identical compositions, the solvent uptake (Δm = m_1_ – m_0_) will decrease as the cross-link density increases. The swollen sample is generally dried under high vacuum for several hours to remove any residual trace of solvent thus allowing determination of m_2_, the mass of polymer incorporated in the chemical network. Four sets of data can be extracted from swelling measurements: (i) the swelling ratio or index of the gel fraction (SI, Figure 8), (ii) the amount of polymer that is not incorporated into the network structure and, therefore, can be extracted as a sol fraction, (iii) conversely the gel content or insoluble fraction (GC, Figure 8), and (iv) the molar mass and composition of the sol fraction (NMR, size exclusion chromatography, but also thermal analyses). The vast majority of measurements rely on gravimetric analysis which require, to be accurate, additional corrections (quantity of material extracted, loss of solvent during weighing, etc.) that are rarely performed.^134^ CANs should demonstrate a high gel content (>90%) in an appropriate solvent. The material should also sufficiently swell (swelling index >50%), enabling the release of unreacted species. For associative CANs, we recommend performing the swelling tests at temperatures above *T*_g_ to reach equilibrium in a rapid and reliable manner. In the case of dissociative CANs, the network connectivity can be altered at high temperature thus affecting the results of swelling measurement. We therefore recommend performing the swelling tests at temperatures where dissociation can be overlooked while ensuring that equilibrium has been reached (e.g., by increasing experiment time). Swelling tests can also be used to determine the average molar mass between junctions (or network density) according to Flory–Rehner’s theory,^135^ but several limitations exist. This model does not take into account network defects such as dangling chains and loops and tends to overestimate the molar mass between cross-link and require the determination of Flory–Huggins interaction parameter between a given polymer/solvent couple. Nevertheless, the swelling index and its variation can be used to qualitatively compare samples with similar chemical compositions.

**Figure 8:**
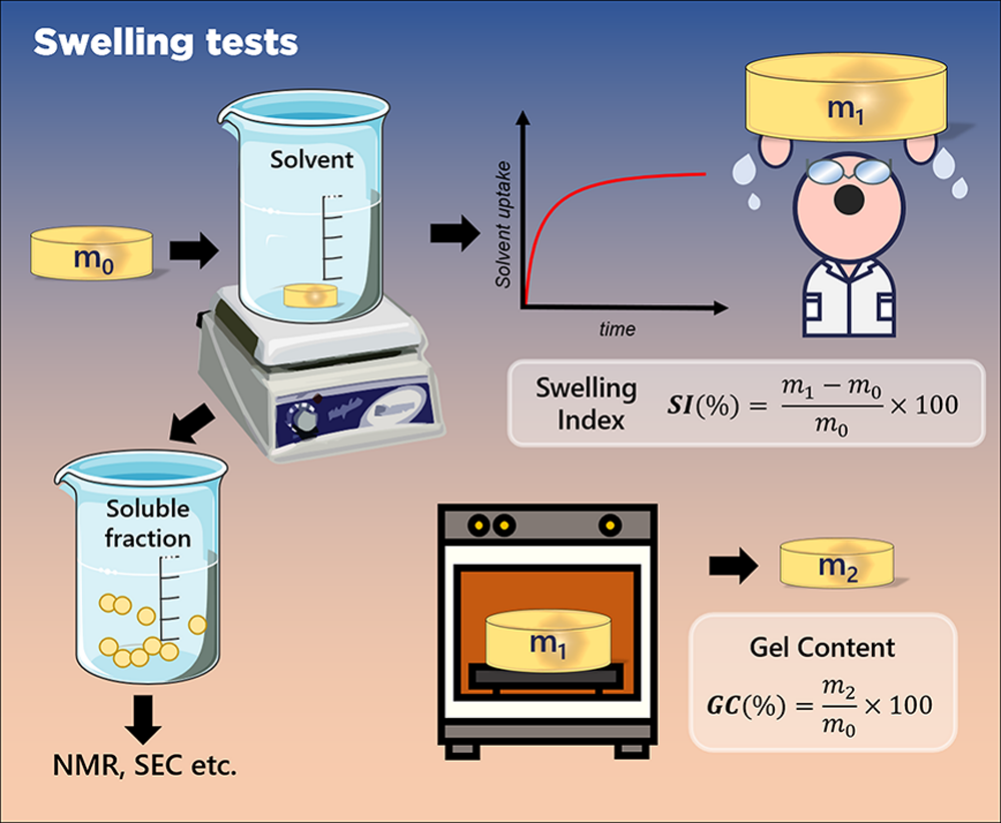
Swelling tests.

### Temperature Window of Analysis

To find out the range of temperature in which the rheological response of a CAN will be solely dependent on the covalent exchange kinetic/mechanism, thermogravimetric analysis (TGA) and differential scanning calorimetry (DSC) should be performed. These thermal characterizations allow determination of the temperature window analysis between the glass transition and the degradation temperature. For amorphous CANs and to avoid artifact due to cooperative segmental relaxations (associated with the glass transition) during rheological measurements, it is advised to perform measurements between [*T*_g_ + 50 °C; *T*_d_ (2%) – 20 °C] (Figure 9). In addition to TGA and DSC, other techniques, such as temperature variable spectroscopy or isofrequency small-amplitude oscillatory shear measurements, should be used to evaluate thermal stability. Indeed, polymers can undergo degradation reactions at lower temperatures without mass loss. These tests provide useful insights on the sample thermal stability, thus giving the temperature range in which, the characterization methods can be performed (Figure 9). Selecting this temperature window ensures that the physical manifestation of the transition from the glassy to rubbery state or the degradation of the material will not interfere with the assessment of the dynamic CAN properties.

**Figure 9:**
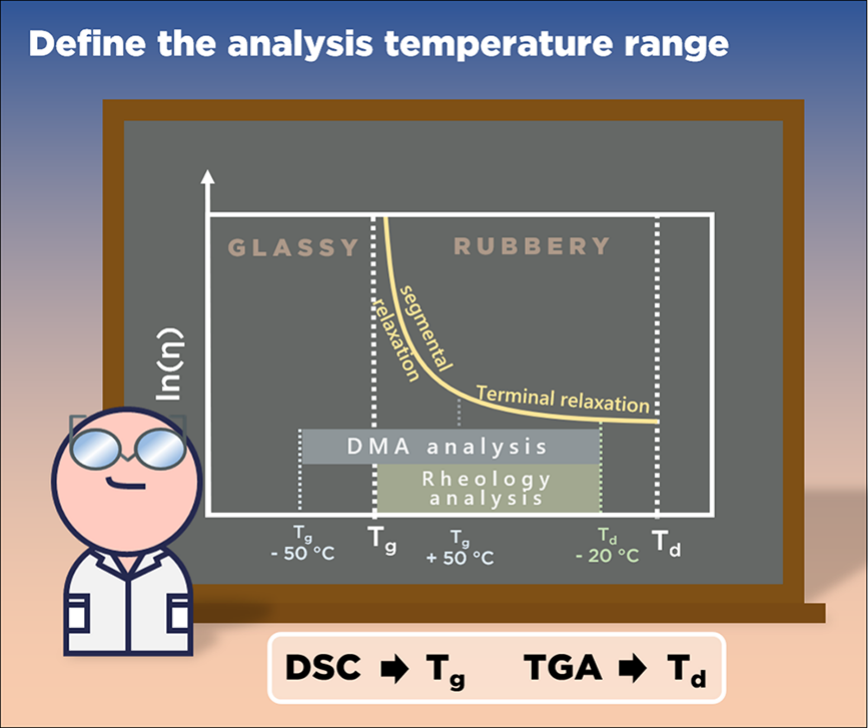
Thermal window of rheological analysis for amorphous CANs.

### Mechanical Tests and Reprocessing Tests

Prior to determining the recyclability or reprocessability of a covalent adaptable network, it is fundamental to know its mechanical properties to define the range of its potential application. Numerous modes of deformations can be employed, depending both on the material state (soft, brittle, gel etc.) and future use in real conditions.^60,136^ For comprehensive books on polymer/composite mechanical properties and characterization, readers can refer to the works of R. F. Landel and L. E. Nielsen. The different modes of deformation presented in Figure 10A include tension, compression, three-point bending, rotational shear and torsion. Tensile and compressive tests are classically employed to determine the mechanical properties of polymers, polymer networks, and composites. By monitoring the stress (σ) and as a function of an applied strain (ε) at a defined strain rate (έ), strain–stress curves allow to evaluate material properties and characteristics such as the Young Modulus (slope at low strain), the ultimate stress and the strain at break (σ _MAX_ and ε _MAX_), and the toughness (area under the stress–strain curve) as depicted in Figure 10B.

**Figure 10:**
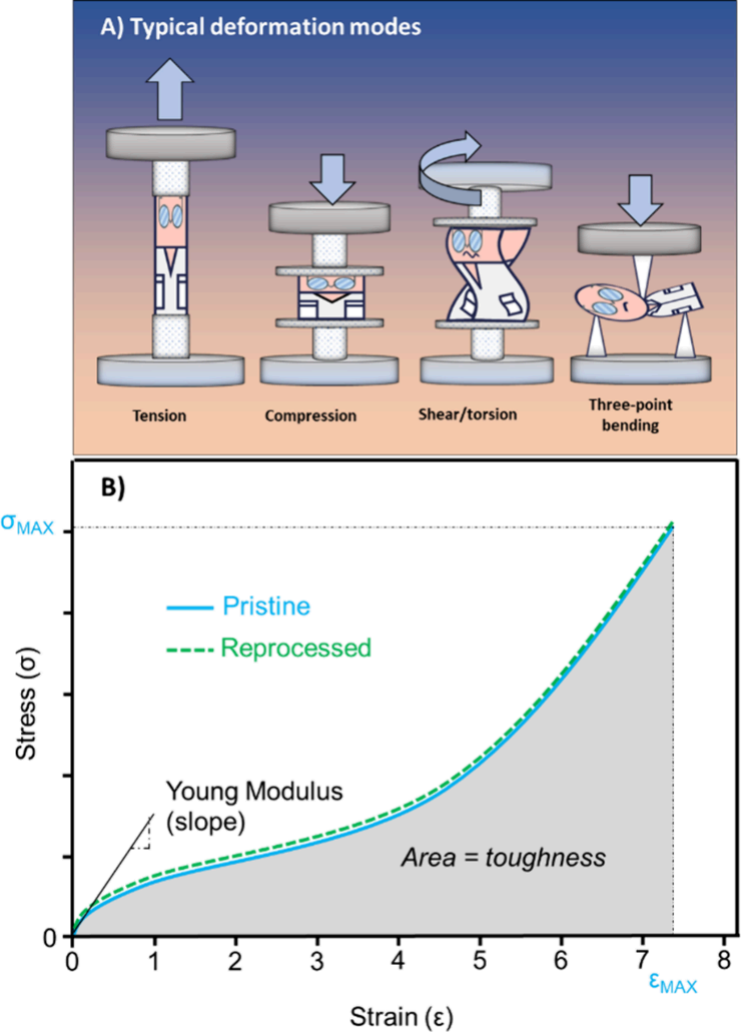
(A) Typical deformation modes used in mechanical tests. (B) Schematic of stress strain experiments to measure the recovery of mechanical properties after reprocessing cycles. Key values include the ultimate strength and stress at break, Young’s modulus is extracted from the slope at origin.

Owing to the presence of exchangeable bonds in a cross-linked material, macromolecules are capable of diffusing and healing damage and defects. Depending on the exchange rate, CANs can thus be reprocessed by compression molding or more rarely by extrusion/injection processes.^22,56,87^ Reprocessing or reshaping tests, commonly performed by compression molding, consist of cutting/grinding the pristine material into small pieces before placing them in a mold and applying a given pressure at a selected temperature. Optimal conditions (time, pressure and temperature) are generally picked out thanks to rheological measurements by the determination of characteristic relaxation time (*vide infra*). The chemical, physical, thermal, and dynamic properties of the reprocessed CANs should be compared with those of the pristine materials. Indeed, as is sometimes observed for thermoplastics, alterations of some properties can occur after several reprocessing cycles. The precise analyses of the reprocessed materials may allow to pinpoint the cause of these modifications of properties (i.e., network rearrangement, thermal degradation, loss of connectivity, secondary reaction or loss of crystallinity, for example).^137,138^ The repeatability of such measurement can be relatively low; it is thus advised to perform mechanical tests at least in triplicate for each material, following the American Society for Testing and Materials (ASTM) norms and recommendations. The characterizations that were performed on the pristine material, such as FTIR, DSC, TGA, insolubility test and DMA, should also be carried out on the reprocessed CANs. Moreover, even if not commonly performed, it could be of interest to check that the dynamic properties of the CANs are not altered by the reprocessing by performing at least one of the analyses described in section Rheological Measurements: A Practical Case (*Vide Infra*). Performing these analyses on the reprocessed material allows precise evaluation of the reprocessability efficiency of these CANs. Reprocessing experiments combined with nonlinear rheology such as tensile test experiments provide valuable and complementary information about the rheological behavior of such material at large deformation, therefore most similar to the conditions of use. Alternatively, the healing ability of CANs could be measured by single lap-shear test.^139^ Thanks to covalent exchanges and chain diffusion at the interface, CAN are able to self-weld, which is impossible for conventional thermosets.

In addition to reprocessability, the presence of exchangeable bonds in CANs allows these materials to be dissolved or depolymerized in a reactive solvent. As mentioned in the Introduction, CANs, just like thermosets, are, by definition, insoluble in a good solvent. However, in the presence of an excess of a reactive solvent (e.g., alcohol, amine, thiol, etc.) which can react with the exchangeable moieties, they may be dissolved (although heating may be needed to perform this dissolution). This phenomenon is particularly useful for the recycling of CAN-based composites. Indeed, in the presence of a reactive solvent, cross-linking dynamic bonds are readily broken which allows to dissociate, collect, and recycle (potentially) both the organic fragments of the CAN matrix and the composite reinforcement components. Specific reviews dealt with the development of CAN composites and the solubility of the polymer resin in reactive solvents.^36,85,140–143^ For instance, the incorporation of disulfide bonding into a polyester matrix for the manufacture of carbon or glass fiber composite allows the dissolution of this polymer matrix in a thiol solution and thus the simple and efficient recovery of the initial fiber.^144^ The dynamic properties of the CAN matrix also allow the overall reshaping of the material after milling.^144–146^ This depolymerization process has been industrialized by Mallinda. This company has developed composites from polyimine matrix and carbon fibers, allowing the shaping of a cross-linked network in a simple way, the repair of damaged materials and the complete dissolution of the composite matrix using imine exchange chemistry.^147^

The case of semicrystalline CANs was less studied and limited to aromatic polyesters (polyethylene terephthalate^148^ or polybutylene terephthalate^149,150^ ) and polyethylene-based CANs.^151,152^ In addition to the glass transition, semicrystalline polymer exhibits a melting temperature (*T*_m_). Linear rheological measurements such as stress relaxation, creep-recovery or small amplitude oscillatory shear (described below) were generally performed at temperature higher than the melting temperature (starting from *T*_m_ + 5 °C to *T*_m_ + 50 °C).^153^ Solid state rheological studies were also investigated below the melting temperature of PBT-based CAN by performing tensile experiments (nonlinear rheology) at various temperature.^150^

## Rheology Basics: Simplest Models, Static vs Dynamic Solicitations and Thermal Transitions

This section offers readers of this tutorial a review of fundamental rheology basics to understand the rheological responses (and associated transitions) of thermoplastics, thermosets, and CANs. Polymer materials, regardless of their topology (thermosets, thermoplastics or CAN), are viscoelastic, i.e. they combine both viscous and elastic responses when subjected to a deformation or a stress. Ideal elastic solids store energy when a strain is applied and return to their original shape if the deformation applied is within the linear domain (*vide infra*). By opposition, an ideal liquid dissipates energy by adopting a new shape. Viscoelastic materials possess both characteristics of elastic solids and of viscous liquids; i.e., they are able to both store and dissipate the energy when subjected to external forces. Polymer responses to applied stress can be divided in three ways: (i) rapid and elastic response (high modulus), which corresponds to the bond stretching and bending, (ii) viscous flow (low modulus), associated with the irreversible slippage of flow units, or (iii) in a rubber-elastic manner characterized by a low modulus but largely reversible slippage of flow units retarded by the internal forces (friction) when flow units orient themselves along the stress axis.

Two main models invented in the 19th century, consisting of dashpots and springs (Figure 11) are useful to describe polymer viscoelasticity despite being limited to amorphous polymers at really low shear stress. For general reviews/books on polymer rheology and viscoelasticity, the reader is directed to reference books of Ferry^154^ and Lenk.^155^ The main difference between the Maxwell relaxation and Kelvin retardation models is that in the Maxwell model springs and dashpots are arranged in series, so they each bear the total stress. In the Kelvin–Voigt model, elements are arranged in parallel, thus distributing the stress among themselves. As polymers are composed of multiple segments with distinct relaxation modes, they exhibited a spectrum of relaxation times which cannot be described by the above-mentioned simple models. The relaxation (τ) or retardation (τ_J_) time is defined as the ratio of viscosity and modulus (η/G) and can be obtained from respectively stress relaxation experiments at constant strain, and creep experiment at constant stress follow by recovery (when the stress is removed). The determination of such parameters and adequate methodology will be further discussed in the section Rheological Measurements: A Practical Case.

**Figure 11:**
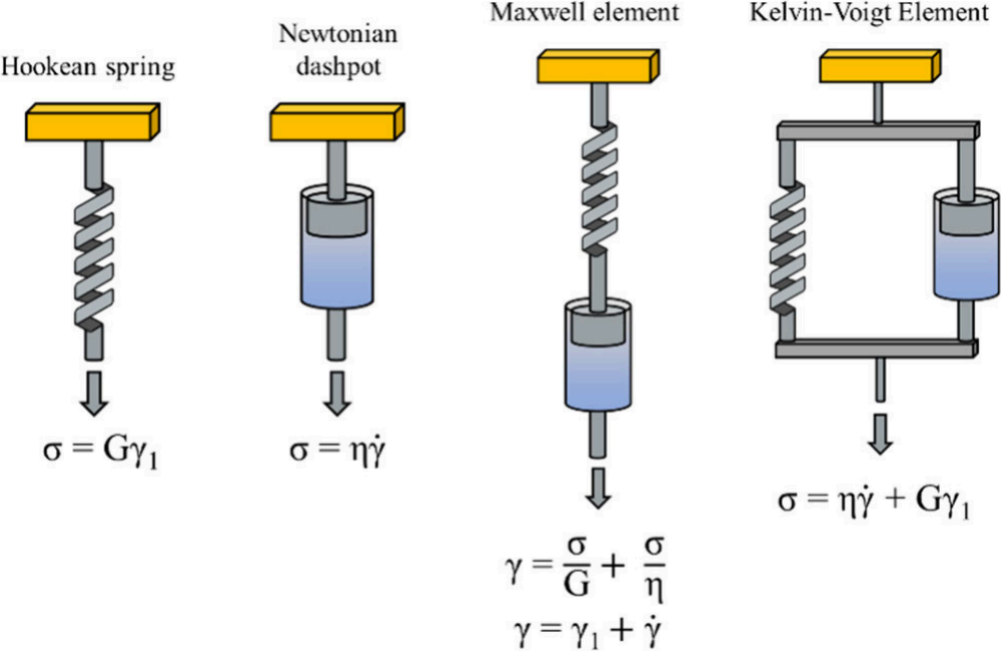
Viscoelastic elements and simplest models, σ = stress; γ = total strain; γ_1_ = strain on the spring; *γ̇* = strain rate, η = viscosity (Newtonian), and G = spring modulus.

Dynamic mechanical analysis (DMA) and small amplitude oscillatory shear (SAOS) generally employed small deformations (γ ≤ 10%) at various frequencies. When performing DMA or SAOS experiments, a sinusoidal strain is applied to the material, allowing us to extract simultaneously both the elastic and viscous contributions (Figure 12A). The storage and the loss moduli, which correspond to the in-phase and out of phase responses, respectively, can be readily separated with dynamic tests. Typical modes of deformation include tension, compression, shear (simple or rotational), three-point bending or torsion. The typical evolution of storage modulus (elastic or shear) as a function of temperature is represented in Figure 12B for each family of polymer.

**Figure 12:**
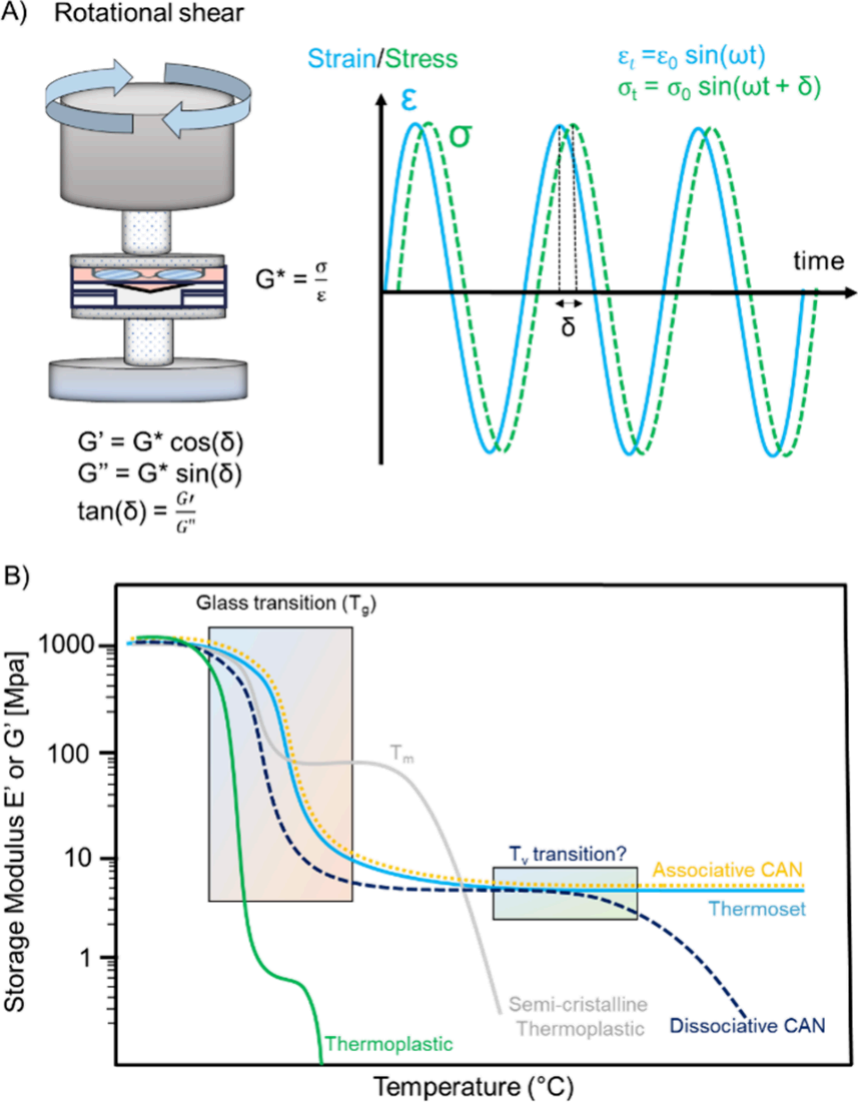
(A) Schematic of small amplitude oscillatory shear experiments, G* = complex shear modulus, G′ = storage modulus, and G″ = loss modulus. (B) Evolution of storage modulus as a function of temperature for thermoplastics (amorphous and semicrystalline) and thermosets.

At temperatures lower than the glass transition temperature, thermoplastics (both amorphous and semicrystalline), thermosets, and CANs, regardless of their chemical nature, structure, or topology, exhibit an almost similar glassy behavior with modulus values of a few GPa. As the temperature increase above *T*_g_, a significant drop in moduli corresponding to the glass transition is observed and different behaviors can be discerned. For thermoplastics, a further decrease in modulus related to their so-called terminal relaxation is observed at higher temperature. In the presence of an amorphous polymer, a rubbery plateau of few kPa (depending on the chain entanglement) can be monitored at high temperature before the terminal relaxation, whereas for crystalline polymers the rubbery plateau is located around several MPa. Several parameters such as the molar mass (and dispersity) or the architecture (linear, branched, cyclic, etc.) of the polymer chains influence the rheological response of thermoplastics resulting from the entanglement of these chains. The permanent cross-linked structure of thermosets results in a rubbery plateau extending over a wide temperature range until material degradation (Figure 12B).^156^ When stress is applied to a chemical network, the so-called elastically effective strands are stretched, thus increasing the free energy of the system (entropic cost). For an ideal network, i.e. without dynamic bonds or defects, existing models predict the elastic shear modulus is directly proportional to cross-link density or the number of elastically effective strands. Two simple models are commonly used to extract the number-average molar mass between strands (or the cross-link density): the affine network model (eq 3) and the phantom network model (eq 4). Knowing the shear (or elastic) storage modulus at *T*_g_ + 50 °C allows us to determine the molar mass of a network strand (M_s_). As mentioned above in the case swelling test, such models do not reflect the reality.

3 $${G^{\prime }}_{\left(T_{g}+50\;\mathrm{{}^{\circ}C}\right)}=\frac{\rho RT}{M_{s}}$$

4 $${G^{\prime }}_{\left(T_{g}+50\;\mathrm{{}^{\circ}C}\right)}=\left(1-\frac{2}{f}\right)\frac{\rho RT}{M_{s}}$$

where G′ is the shear storage modulus at *T*_g_ + 50 °C, R is the ideal gas constant, *T* is the absolute temperature, ρ is the network mass density, and *Ms*, the number-average molecular weight of a network strand between junctions, *f* is the functionality of junctions introduced in the phantom model to consider the statistical distribution of junctions within the networks.

Due to the occurrence of covalent exchanges, new and hypothetical transitions have been also put forward for CANs. While a thermal transition from gel to sol has been already observed in the case of dissociative CANs, the constant cross-link density of associative CANs or vitrimers makes the hypothetical transition from a thermoset to a thermoplastic behavior more complex to characterize.

### The Case of Topology Freezing Temperature

In addition to *T*_g_ and *T*_m_, another transition named topology freezing temperature (T_v_) was introduced in the seminal work of Leibler’s group.^16^ This transition was meant to describe the *thermoset to thermoplastic* transition. The T_v_ is generally determined by an extrapolation of the Arrhenius viscosity plot to a lower temperature (ln­(η) vs 1/T, *vide infra*). A viscosity of 10^12^ Pa·s was chosen as an arbitrary value to characterize the onset temperature when covalent exchange reactions become relevant in the experimentally observed time frames. Indeed, if the viscosity is higher than 10^12^ Pa·s, the material is considered for most applications as stable, whereas for viscosity below this limit value, material flow must be considered. In a recent contribution, Klinger et al. demonstrated by combining dilatometry experiments and temperature-modulated optical refractometry that vitrimer creep or flow ultimately stops at the *T*_g_ which seems to indicate that no other transitions exist (apart from operational artifact).^157^ Even if the relevance of the T_v_ can be discussed,^31^ its comparison with the other characteristic transition temperatures (*T*_g_ and *T*_m_) enable to determine the temperature range within which an Arrhenius behavior can be expected. As mentioned above, exchange reactions occurring in a polymer matrix are limited not only by the kinetics of the reaction itself but also by the polymer chain mobility. Hence, below *T*_g_ (or *T*_m_ if exchangeable groups are present in the crystalline regions) exchange reactions are restrained because of the reduced chain mobility in the glassy state. From this perspective, two cases can be discerned (Figure 13):

- *T*_g_ < T_v_: In this case, the viscosity evolves according to the Williams–Landel–Ferry (WLF) model for temperature comprised between *T*_g_ and T_v_, as typical elastomer. At temperature above T_v_ covalent exchanges start to significantly occur and viscosity decreases according to an Arrhenian behavior.
- *T*_g_ > T_v_: In this case, viscosity remains high and stable below *T*_g_. However, above *T*_g_, viscosity rapidly decreases according to the WLF model because of the significant change of chain mobility caused by the exchange reaction which becomes the predominant phenomenon and dictates the evolution of viscosity with temperature.

**Figure 13:**
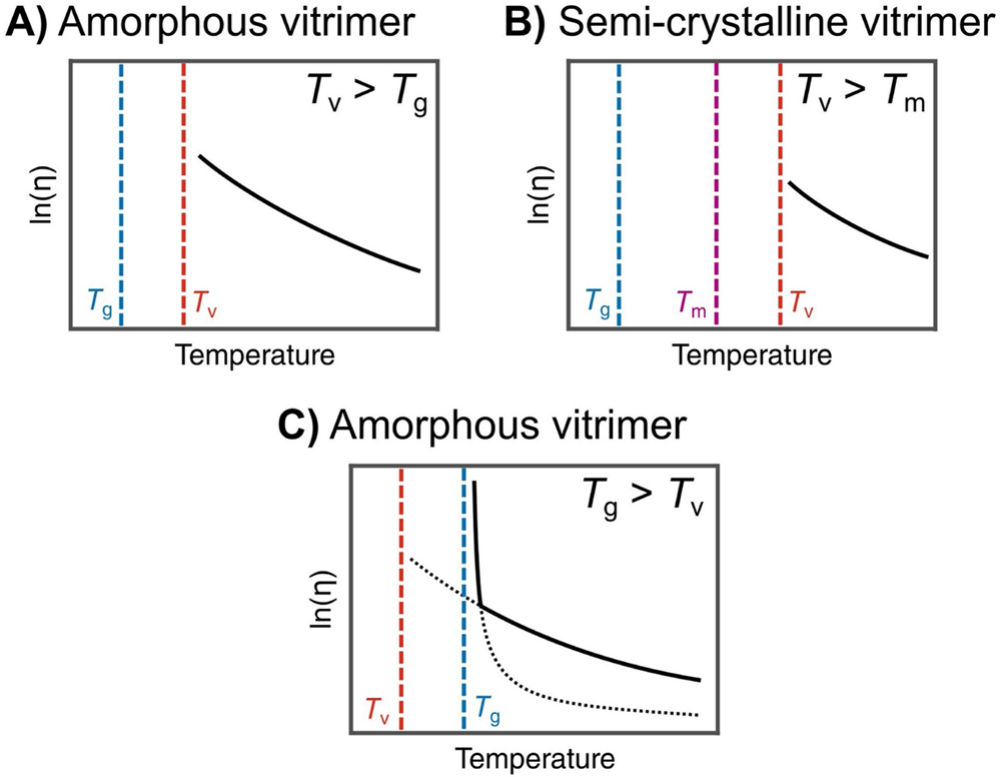
Typical viscosity evolution with temperature according to the T_g_, T_m_, and T_v_ values. Reproduced with permission from ref 8. Copyright 2020 Elsevier.

Another technique allowing the determination of T_v_ is based on the fluorescence of AIE (aggregation-induced emission)-luminogens (Figure 14).^158^ For example, the incorporation of tetraphenylethene (TPE) in CANs, without alteration of thermal or mechanical properties, resulted in drastic changes of fluorescence intensity with temperature. Below T_v_, the AIE molecule is restricted to intramolecular motion while above T_v_, the activated intramolecular rotations of AIE decay the energy of the excited state and weaken its fluorescence emission. The determination of T_v_ by this method was independent of the catalyst loading and heating rate. However, the temperature determined using TPE did not correspond to the definition of the T_v_ but was rather associated with the activation of the exchange reactions.

**Figure 14:**
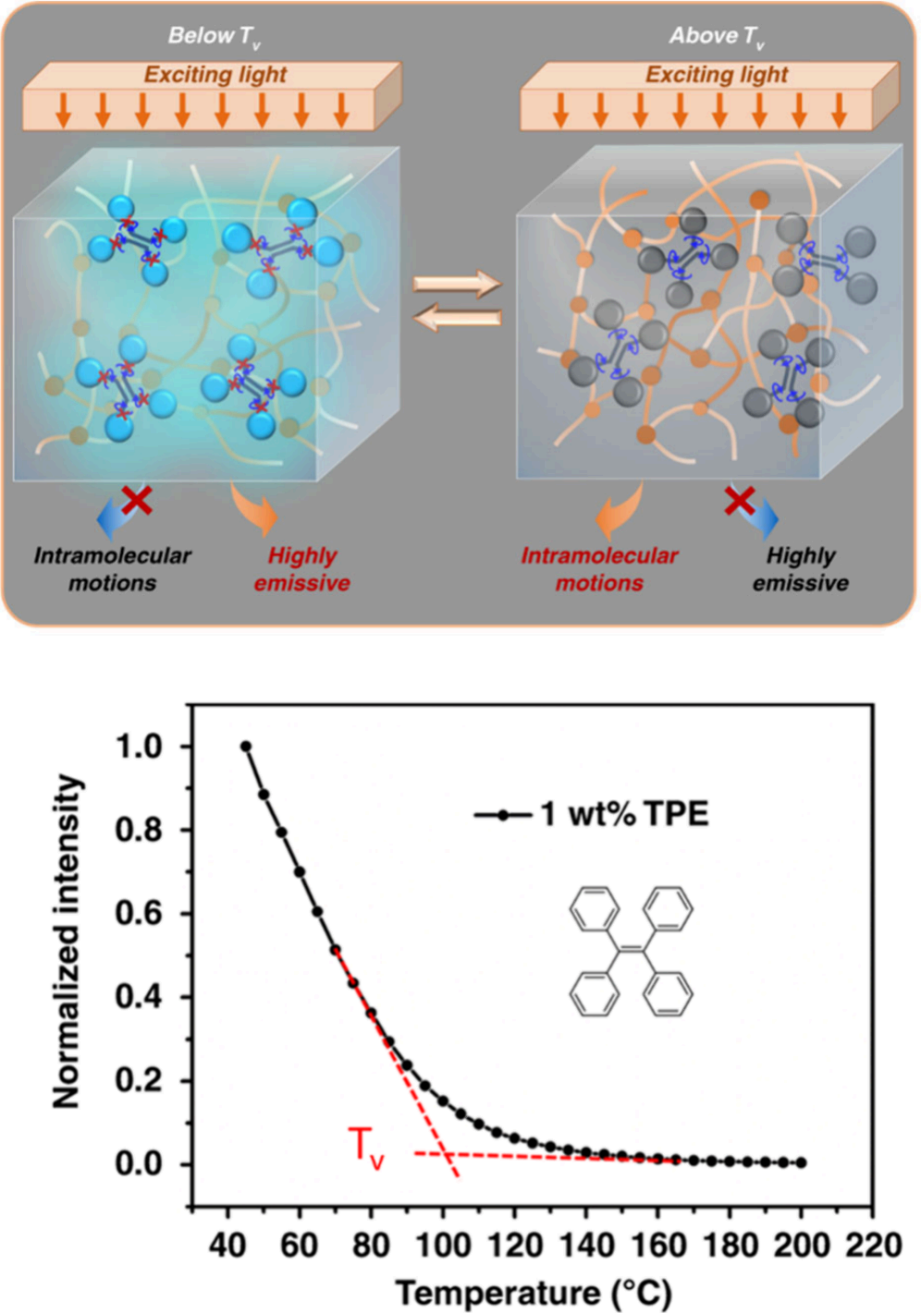
Schematic representation of the AIE-molecule behavior as a function of temperature (top) and the evolution of normalized fluorescence intensity measured at 470 nm as a function of temperature. Bottom panel adapted from ref 158. Copyright 2019 Springer Nature.

## Rheological Measurements: A Practical Case

Three rheological analyses, depicted in Figure 15, are commonly used to characterize and compare the flow behavior of CANs. Stress relaxation, creep/recovery, and small amplitude oscillatory shear (or frequency sweep) allow us to determine respectively the characteristic relaxation time (τ) and retardation time (τ_J_) and the crossover frequency (ω_G′=G″_). In the following sections, these methodologies, the associated physical quantities and their relationship as well as guidelines to choose the most relevant analysis are described. For the following rheological characterizations, we selected a CAN based on trifluoromethylated *N,S*-acetal^120^ that our team recently published in *Macromolecules*. The material is readily obtained by reacting a trifunctional thiol with a bifunctional trifluoromethylated imine yielding a CAN with low *T*_g_ (−10 °C) and T_d5%_ of 200 °C.

**Figure 15:**
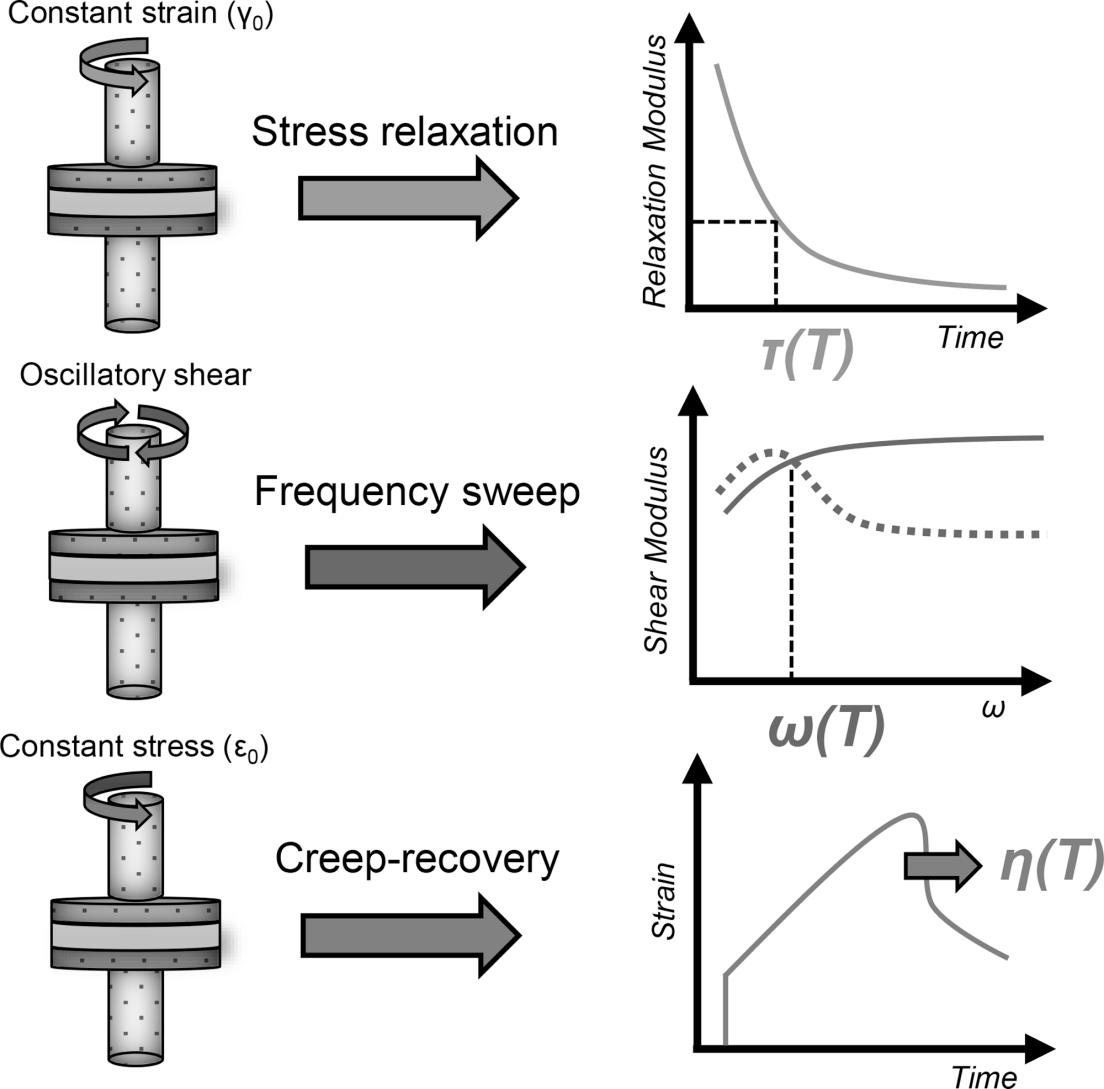
Three main rheological characterizations and associated physical quantities employed for the characterization of CAN dynamic properties.

Rheological analyses require mechanical characterization devices such as a rheometer (strain or stress controlled) or dynamic mechanical analysis (DMA) apparatus. The rheometer has been preferred to the DMA as shear rheology is generally recommended for molten/liquid samples (to characterize material’s flow) while dynamic mechanical analysis is more appropriate for free-standing films that are too stiff for shear rheometers. From a practical point of view, it is generally advisable to work on a rheometer (strain controlled) equipped with an 8 mm plane/plane geometry, preferably textured (striated or sandblasted) to minimize slippage during analysis. A steady normal force is generally applied in the range of 0.5 to 5 N (preferentially 1 N), thus ensuring adequate contact between the samples and the geometry. Torsion mode is also appropriate even if it has been rarely employed for vitrimer characterization.^159^ The determination of the material’s linear viscoelastic domain is a prerequisite to all rheological analyses listed below. because it allows an easier evaluation of the material response. In linear rheology experiments, the stress/strain applied to the materials is theoretically infinitesimal, meaning that the molecular structure of the material is unaffected during the experiment. Small deformations of 0.5 to 10% are generally employed to ensure that the material response is high enough to be measured without disturbing the molecular landscape. In order to determine the linear viscoelastic domain, a strain or stress sweep analysis must be performed beforehand at the desired temperatures (at least at both extrema of the analysis window). The dynamic properties of CANs should be analyzed from at least a temperature 50 °C above the glass transition temperature. This precaution allows to separate the phenomena related to the glassy to rubbery transition (which can be modeled with Rouse or sticky-Rouse models^160^) from those related to exchange reactions enabling to simplify their rheological evaluation.^161,162^ In Figure 16, strain amplitude sweep experiment at 100° and 150 °C for CF_3_-*N, S*-acetal CAN showed the evolution of shear loss and storage modulus between 0.01 and 50% strain. The critical strain (γ _crit_) was determined as the onset of G′ drop, e.g., γ _crit,100 °C_ = 1% at 100 °C and γ _crit,150 °C_ = 10% at 150 °C. A maximum strain of γ _crit_/2) was employed during stress relaxation experiments to ensure that the applied strain was within the linear viscoelastic domain.

**Figure 16:**
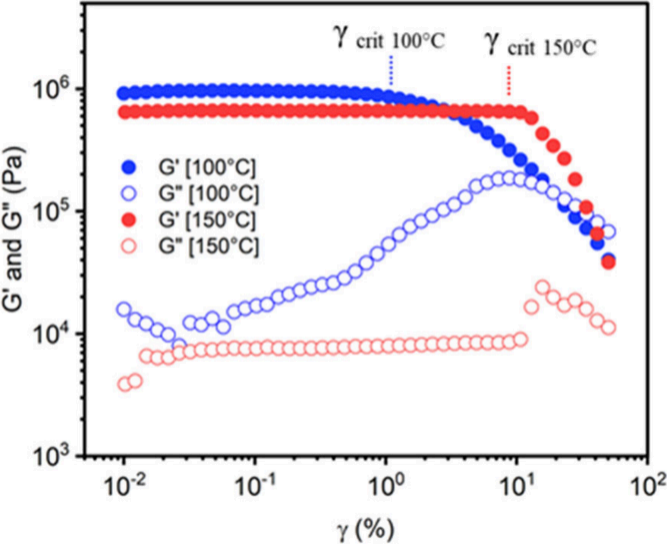
Strain amplitude sweep experiment at 100 °C (in blue) and 150 °C (in red) between 0.01 and 50% strain at 1 rad·s^–1^ for CF_3_-*N,S*-acetal CAN. Reproduced from ref 120. Copyright 2024 American Chemical Society.

In the linear viscoelastic domain, the storage modulus (G′ and E′) are independent of strain while the loss modulus (G″ or E″) are independent of the strain rate. In addition, the complex modulus G*_ω_ or E*_ω_ must follow a sinusoidal law with a frequency identical to that of the deformation. It is also important to monitor the time required to apply the strain as a delay of 0.1 to a few seconds is generally needed to achieve the desired deformation (depending on the rheometer device), meaning that constant strain/stress experiments are not suitable for ultrafast relaxation (τ ≤ 10 s).

### Stress Relaxation

Stress relaxation experiments have been the method of choice to determine the characteristic time scale of CANs flow behavior. During a stress relaxation test, an instantaneous and constant strain (within the linear domain) is applied to the sample and the relaxation modulus (G_(t)_) is followed as a function of time. Isothermal non-normalized stress relaxation experiments (100–150 °C) of CF_3_-*N, S*-acetal are presented in Figure 15A. The applied stress required to maintain the deformation of the sample decreases as a function of time due to the network reorganization. Different models have been applied to extract the characteristic relaxation time (τ) from experimental curves. In an ideal system with a single relaxation mode, the relaxation modulus decreases according to the Maxwell model (eq 5 and Figure 17):

5 $$G\left(t\right)=G_{o}\exp\left(-\frac{t}{\tau }\right)$$

where t is time, G_0_ is the initial relaxation modulus and τ is the characteristic relaxation time. The value of τ obtained by Maxwell modeling therefore corresponds to a time (at a defined temperature) for which the value of the relaxation modulus is equal to 1/e or ∼ 37% of its initial value (G_0_).

**Figure 17:**
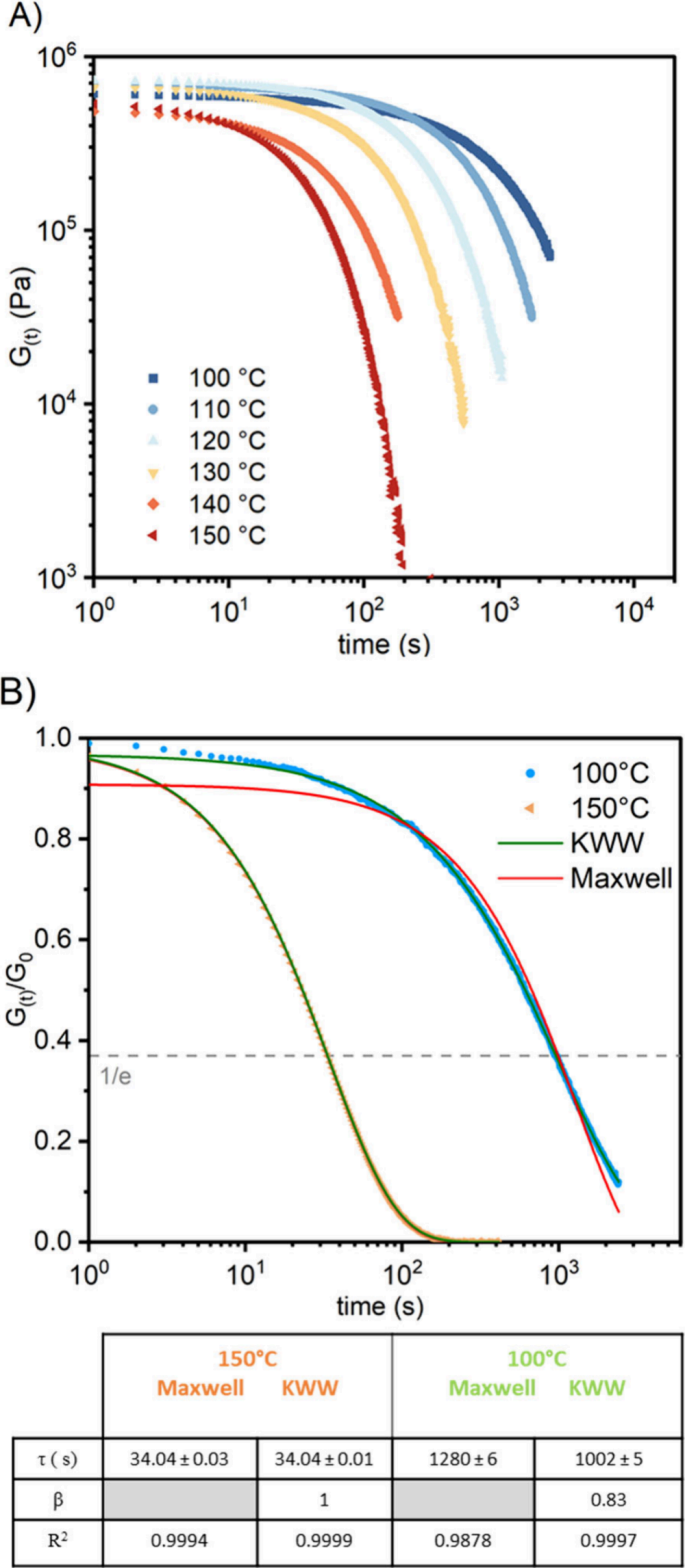
Example of experimental stress relaxation (8 mm sandblasted parallel plate, γ = 0.5%). (A) Nonnormalized isothermal SR experiments between 100 and 150 °C. (B) Curves with different distribution of relaxation times fitted using the Maxwell model (red curve) and by the KWW model (green curve). Reproduced from ref 120. Copyright 2024 American Chemical Society.

However, as material are far to be ideal and contain networks defects (loops dangling chains etc.) and consequently multiple relaxation modes, fitting the Maxwell equation generally failed.^16,74,75^ Taking into account the multimodal distribution of relaxation modes, the stress decay is more accurately fitted by using a stretched exponential function in the so-called Kohlrausch–Williams–Watts (KWW) model (eq 6 and Figure 17B):^163^

6 $$G\left(t\right)=G_{o}\exp\left(-{\left(\frac{t}{\tau^{*}}\right)}^{\beta }\right)$$

in which β is the stretching exponent and τ* the relaxation time obtained from the fitting with a stretched exponential. It has to be noted than for both Maxwell and KWW models, at t = τ or τ*, G_t_/G_0_ = 1/e (0.37). In order to precisely determine τ* and β, relaxation curves should ideally demonstrate a complete relaxation (G_t_/G_0_ < 0.02). Complete relaxation also proved that the material can fully relax stress (and thus does not contain percolated permanent cross-links) and improve the precision of the fitting. The stretching coefficient (β), whose value ranges from 0 to 1 represents the distribution of relaxation modes within the CAN. The closer the value is to 1, the closer the model is to the Maxwell model, *i.e*., to a single relaxation mode (eq 1). As an example the recorded relaxation of CF_3_-*N,S*-acetal networks at 150 °C, were fitted using both Maxwell and KWW models showing identical results (β = 1). At lower temperature; (β = 0.83), discrepancies were observed with extracted relaxation times τ = 1280 s (Maxwell) vs τ* = 1002 s (KWW). The single Maxwell failed to catch the broader spectrum of relaxation times.

Isothermal stress relaxation experiments give access to the characteristic relaxation time (τ*) which is dependent on a series of parameters, such as the exchange reaction mechanism, the exchange reaction rate, the temperature at which the test is carried out, the availability of exchangeable groups within the network, and the cross-link density and nature (polarity) of the polymer network. Data are still missing to fully understand how all of these related parameters influence the viscoelastic behavior of CANs. For instance, an increase in cross-link density was demonstrated to slow down the exchange rate in the case of dioxaborolane associative CANs^19^ whereas in the case of vinylogous urethane, increasing the cross-link density leads to shorter relaxation times.^39^ A variation of cross-link density implies the modification of two parameters playing an opposite role in the exchange rate: the concentration of exchangeable bonds and the chain mobility. Indeed, a rise of cross-link density induces a reduction of the chain mobility but might bring exchangeable functions closer to each other.

In some cases, multiple mode relaxation profiles can be caused by the occurrence of several reactions (or side reactions), which proportion can change during the network rearrangement.^164^ For example, in the case of transesterification vitrimers prepared by epoxy-acid reactions, initially the hydroxyl groups are mostly secondary, but after transesterification a primary alcohol is produced, thus generating heterogeneity in the types of exchangeable functions.^102,165,166^ Similarly, phase separated systems, in which exchangeable bonds are heterogeneously distributed in different immiscible phases, exhibit more complex (often stretched) relaxation modes.^167,168^ In addition to the evolution of the relaxation time with temperature, the dependence of the initial relaxation modulus (G_0_) on temperature could also provide information. As mentioned above, for dissociative exchange, the cross-link density decreases with temperature, resulting in a decrease of G_0_. Thus, relaxation tests carried out at different temperatures can, to some extent, provide evidence on the exchange mechanism. This observation has been used, for example, to support the conclusion that transalkylation of triazolium occurred via a dissociative mechanism^169^ or that a transamidation exchange proceeded via the formation of an imide intermediate.^90^ It is important to note, however, that if G_0_ remains stable with temperature, it is not possible to conclude the nature of the mechanism. Indeed, in some dissociative systems for which the association is significantly faster than the dissociation reaction, the reduction of cross-link density could be negligible in the studied temperature window and therefore not observable. To observe this variation of G_0_ with temperature, it is recommended to plot the non-normalized relaxation curves in log (G_T_) vs log (time) plot.^20^

### Creep and Recovery

The creep recovery test is carried out in two stages. In the first stage, a static stress (σ = constant) is applied to the material, and in the second stage, the stress is released (σ = 0). The strain (γ­(t)) is monitored over time. In the case of thermosets, only elastic deformation (reversible) is observed during the first instants of stress application, whereas for thermoplastics, elastic deformation is followed by plastic deformation (irreversible). As thermoplastics above their *T*_g_ are viscoelastic liquid, the deformation (γ) increases constantly reaching a constant strain rate (with γ ∝ time). During recovery, the elastic deformation is recovered, while the plastic deformation is retained by the material. In the case of CANs, plastic deformation is also observed at temperatures where covalent bond exchange takes place to a sufficient extent.^20,170^ Indeed, the dynamics of the network upon heating allows the material to permanently deform under the application of a stress.^19^ The creep/recovery test was mainly performed on CANs to access the temperature range in which the material behave as classical thermosets (with limited creep), by determining the temperature from which the material start to flow significantly.^19,22,99,171,172^ However, additional information about the mechanical and dynamic properties of the materials can also be obtained from these plots. For example, creep recovery experiments were performed between 70 and 120 °C for the CF_3_-*N,S*-acetal CAN (Figure 18). During the plastic deformation stage, the compliance can be related to the zero-shear viscosity (η_0_) by eq 7:^20^

7 $$J\left(t\right)=\frac{t}{\eta_{0}}+J_{0}$$

where J_0_ is the compliance value corresponding to the elastic deformation. J_0_ can be either (i) estimated from the intercept of the viscosity linear regression or (ii) calculated from the ratio of the strain at the end of the creep minus the strain after recovery (noted Δγ in Figure 18A and the applied stress (in the example 2 kPa).

**Figure 18:**
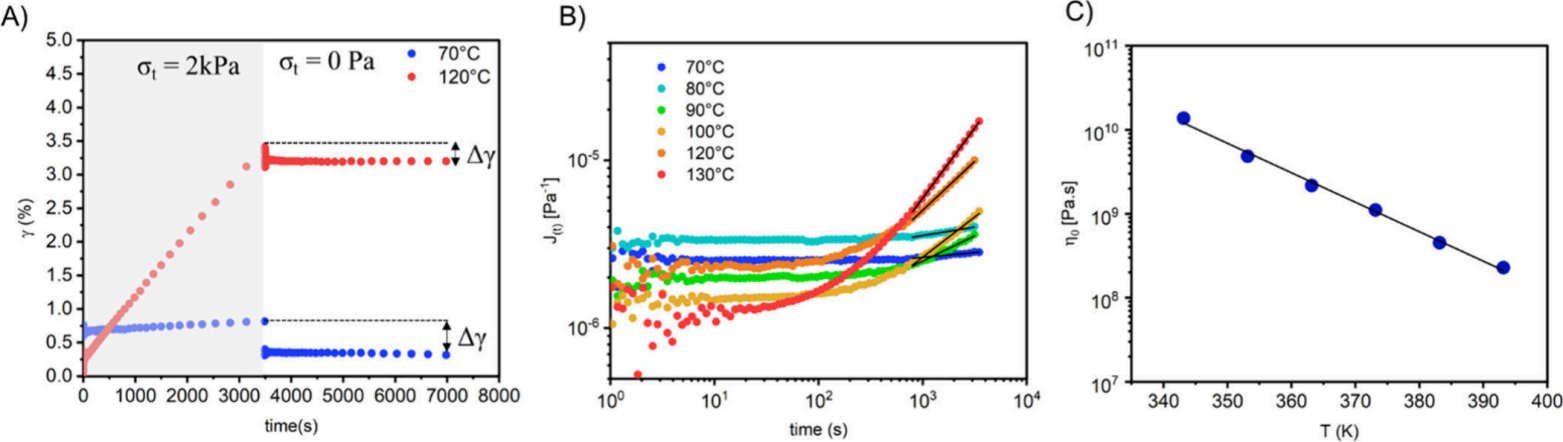
(A) Creep and recovery tests for CF_3_-*N,S*-acetal CANs carried out between 70 and 120 °C (only 70 and 120 °C are presented for clarity). (B) J_(t)_ vs time as a function of temperature (log/log scale); viscosity is extracted from the slope in the steady state regime. (C) Zero-shear viscosity (Pa·s) as a function of temperature (K). Reproduced from ref 120. Copyright 2024 American Chemical Society.

The retardation time (τ_J_) can then be determined via eq 8:

8 $$\tau_{J}={\eta_{0}}^{*}J_{0}$$

Moreover, for temperatures above *T*_g_ + 50 °C, the value of J_0_ is only weakly dependent on temperature for associative CAN, i.e. with a constant cross-link density. Indeed, the elastic deformation, which is a physical representation of the rubbery character of the polymer, only hardly evolves if at all above *T*_g_ + 50 °C. Therefore, η_0_ and τ_J_ have a similar evolution with temperature in the case of associative CANs.^19^ In contrast, an increase in J_0_ with temperature is characteristic of a dissociative exchange mechanism. A typical creep experiment is depicted in Figure 18B for the CF_3_-*N,S*-acetal network, a dissociative CAN.

In addition to the creep/recovery test performed at a constant temperature, dilatometry has also been used to study CANs. In this test, a constant stress is applied to the material during a temperature ramp and the resulting deformation is measured.^16,20,74^ The deformation is linear with temperature, as long as the exchange reactions are not significant. However, beyond a critical temperature, the deformation no longer evolves in a linear fashion, and the material is then considered to be dynamic. This technique affords an approximation of the temperature at which exchanges in the polymer matrix become significant. However, dilatometry has not been widely used for CANs analyses because the temperature of the regime change is linked to the speed of the temperature ramp.^173^

### Small Amplitude Oscillatory Shear (SAOS)

Terminal relaxation of CANs can also be evaluated by small amplitude oscillatory shear experiments (SAOS). This experiment consists of recording the complex modulus (G*) during the dynamic solicitation (*vide infra*) of the sample at a fixed strain (within the viscoelastic linear domain) and varying frequency (generally from 0.01 to 100 rad·s^–1^ depending on the apparatus).

According to the time–temperature superposition principle, the behavior observed at low frequency is identical to the behavior that would be observed at high temperature. The application of this principle thus allows the study of the dynamics of CANS at moderate temperatures. For a given temperature, the crossover of G′ and G″ is representative of network creep (Figure 19), and the frequency at which this crossover occurs is directly related to the relaxation time by the following equation: ω_G′=G″_ = 1/τ*.^163^ Thus, the relaxation times extracted from the crossover frequency values follow a similar evolution to the one extracted from stress relaxation at high temperature.^174^ Ideally, the frequency sweep analysis should be performed using frequencies in random order to limit measurement errors and hence separate frequency and run time impacts.^175,176^ Similarly to the observation made on the variation of G_0_ with temperature, it is possible to follow the variation with temperature of the value of G′ at the plateau. The same logic can be applied thus providing clues about the nature of mechanism (i.e., associative or dissociative) involved in the covalent exchange reaction.^177^ In the example chosen here, the dissociative nature of *N,S*-acetal exchanges is characterized by a strong decrease of G′ as the temperature increases (Figure 19).

**Figure 19:**
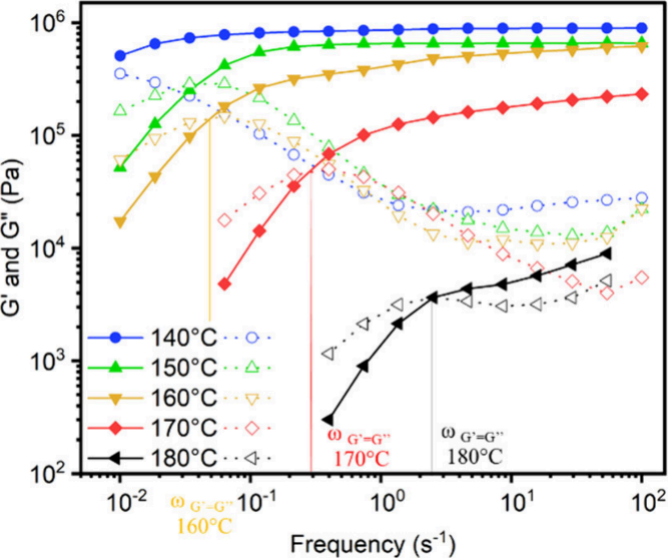
Isothermal small amplitude oscillatory shear (γ = 1% from 0.01 to 100 rad·s^–1^) experiments at different temperatures from 140 to 180 °C for the CF_3_-*N,S*-acetal CAN. Reproduced from ref 120. Copyright 2024 American Chemical Society.

### Which Analysis Method to Use?

From the description of the different analyses, it became clear that the main results of each analysis (τ*, η_0_ or ω_G′=G″_) are intimately related.^178^ Nevertheless, the choice of the analysis methods must be adapted to the characteristic relaxation time of the material (Table 1). Indeed, SAOS experiments allow the determination of very short relaxation times, whereas stress relaxation tests can cover a wider range of relaxation times. The creep and recovery test allows one to determine all types of relaxation times but is generally only used for long relaxation times that are difficult to observe using stress relaxation tests. The limited use of the creep and recovery tests is also related to its accuracy, which is considered to be lower than that of the other two analytical techniques. Nevertheless, as highlighted by Ricarte et al.,^62^ the reporting of non-normalized measurements is required for all the analyses. Indeed, they demonstrated that by using only normalized stress relaxation, a relaxation time of 0.5 s could be determined using the 1/e criterion, whereas this was not representative of the real dynamic of the materials they studied. In their case, creep analysis enabled to better characterize the thermal behavior of the material as relaxation curves could not be fitted with Maxwell or KWW models.^20^ It thus appears that rheological analysis methods are complementary. Even though dynamic parameters are related, their individual determination by different analytic methods ensures the validity of the measurements.

**Table 1:** Summary of the Characteristics of Each Analysis

Analytical method	Key results	Analysis duration (h)	Range of relaxation time evaluated (s)	Complementary information on the mechanism
Stress relaxation	τ*	1–8	10^–1^–10^4^ ^20,165,166,169^	Evaluation of G_0_
Creep/Recovery	τ_J_; η_0_	1–2	10^2^–10^5^ ^19,20,170^	Evaluation of J_0_ (low precision)
SAOS	ω_G′=G″_	0.5–10	10^–3^–10^–1^ ^163,174–176^	Evaluation of G′ on the rubbery plateau

### Complementary Analyses

With the rapid development of CAN chemistry, original characterization methods have been investigated to evaluate their dynamic properties. For example, broadband dielectric spectroscopy (BDS), which uses the material response to an electrical perturbation field over a range of frequencies (10^–2^–10^6^ Hz) and temperature usually provides information on supramolecular systems and molecular motions, has recently been used with CANs.^179–183^ The evaluation of the imaginary part of the permittivity highlights a specific dielectric relaxation which can be associated with the dynamic bond exchange. Indeed, in comparison to the other relaxations observed on thermoset materials, this dielectric relaxation is associated with cooperative molecular motions and is only observed for temperature at which bonds exchange occur significantly. The additional study of the DC conductivity—component of the real conductivity which is independent of the frequency—provides information on the nature of the mechanism involved in the bond exchange since a direct correlation can be established between viscosity and DC conductivity.^179^ *In situ* temperature sweep FTIR analysis is another underused and promising technique to follow the chemical evolution of CANs with temperature. It has been employed so far on dissociative CANs. Indeed, the dissociation of chemical bonds can be followed by the disappearance or appearance of specific vibrational bands in FTIR spectra. This technique has for instance been used to demonstrate the formation of imide bonds in transamidation CANs.^90^ In addition, for vitrimers, the transition state can in theory demonstrate a characteristic band and thus provide knowledge of the exchange steps. Spectroscopic analyses also allow us to monitor and characterize the occurrence of side reactions and their impact on the chemical network composition and structure. As a typical example, our group recently reported the impact of catalyst on the exchange reaction in polyhydroxyurethane-based CANs.^184^ The dissociative nature of the exchange demonstrated thanks to the evolution of shear storage moduli G′ (SAOS experiments, Figure 20A) with temperature. The occurrence of side reactions was highlighted by analyzing the specimens used for rheology experiments by infrared spectroscopy (ATR mode, Figure 20B. The appearance of signature vibration bands corresponding to cyclic carbonate, primary amine (monomer functions), and urea (side products) confirmed both the dissociative mechanism nature already observed in SAOS experiments and the formation of side products (irreversible bonds under these conditions). Jourdain and co-workers also combined thermal treatment and spectroscopic analysis (X-ray photoelectron spectroscopy) to grasp the network composition at high temperature. By quenching a dynamic network based on triazolium cross-links in liquid nitrogen, benzyl iodide groups (dissociative form) could be detected thanks to the diagnostic energy bands of iodine atoms in such moiety­(I_3d5/2_).^169^ The development of new characterization methods such as low-field rheo-NMR^185^ and new theoretical models^186,187^ will provide new tools to respectively characterize and predict the rheological behavior of CANs.

**Figure 20:**
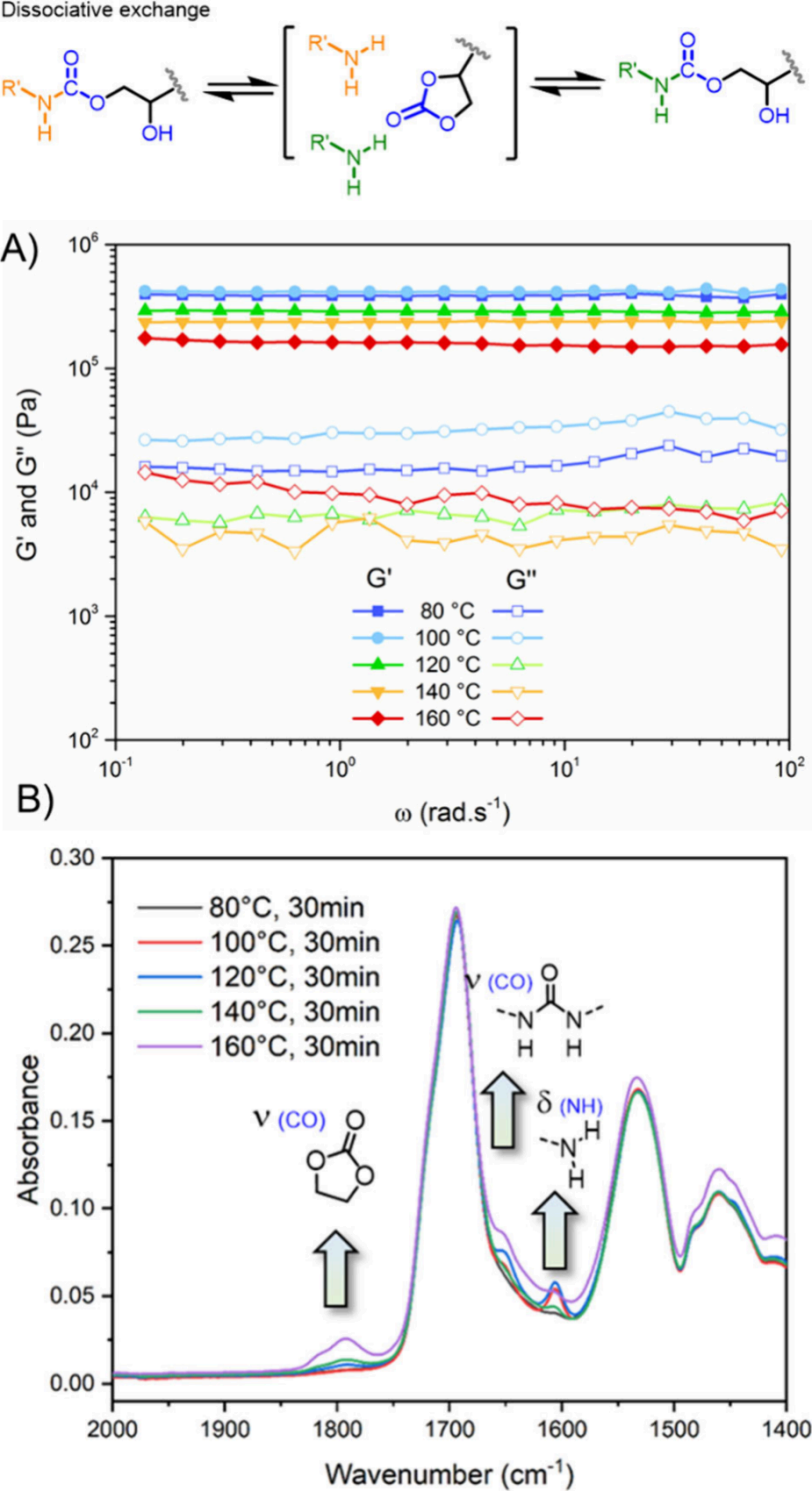
Dissociative exchange mechanism in PHU-CAN catalyzed by *N,N*-dimethyl aminopyridine. (A) SAOS experiments and (B) ATR-IR spectra after SAOS. Reproduced with permission from ref 184. Copyright 2023 Royal Society of Chemistry.

## Suitable Models for Temperature Dependency of CAN Dynamic Properties

In general, the analyses presented above are carried out at different temperatures, thus making it possible to determine the evolution of dynamic properties with temperature. In order to get a complete evaluation of the thermal properties of the CANs and identify possible complex mechanisms, it is highly recommended to examine a wide temperature window.^163^

### Arrhenian Behavior

Similarly, to silica glass, the viscosity of an associative CAN follows Arrhenian-type temperature dependence, as noted in eq 9. This behavior led Leibler’s team to name these materials *vitrimers*.^16^

9 $$\ln\left(\eta_{0}\right)=\frac{E_{A}}{RT}+\ln\left(\eta_{\infty }\right)$$

where E_A_ is the flow activation energy in J mol^–1^, T is the temperature in Kelvin and R is the ideal gas constant (8.314 J·mol^–1^·K^–1^)

Moreover, as discussed in the previous sections, τ* is proportional to η_0_ solely in the case of associative systems (G_0_ ≈ constant at T ≥ *T*_g_ + 50 °C) and its evolution with temperature also follows an Arrhenius law.^19^ By separately measuring the evolution of η_0_ and τ with temperature on epoxy-acyl (transesterification-based) vitrimers, Leibler’s team determined flow activation energies of 80 and 88 kJ mol^–1^ respectively, thus confirming that these two parameters evolve concomitantly.^16,169^ In Figure 21, the characteristic relaxation and retardation times for CF_3_-*N,S*-acetal CAN extracted from creep (70–120 °C), stress relaxation (100–150 °C) and SAOS (150–180 °C) experiments were plotted in a ln­(τ) vs 1/T master curve. Regardless of the method employed, the flow activation energy measured gives relatively similar values with E_A_ = 102.3–127.2 kJ mol^–1^. This result suggests that in the range of temperature studied (from 70 to 180 °C) the relaxation time follows an Arrhenian-type behavior.

**Figure 21:**
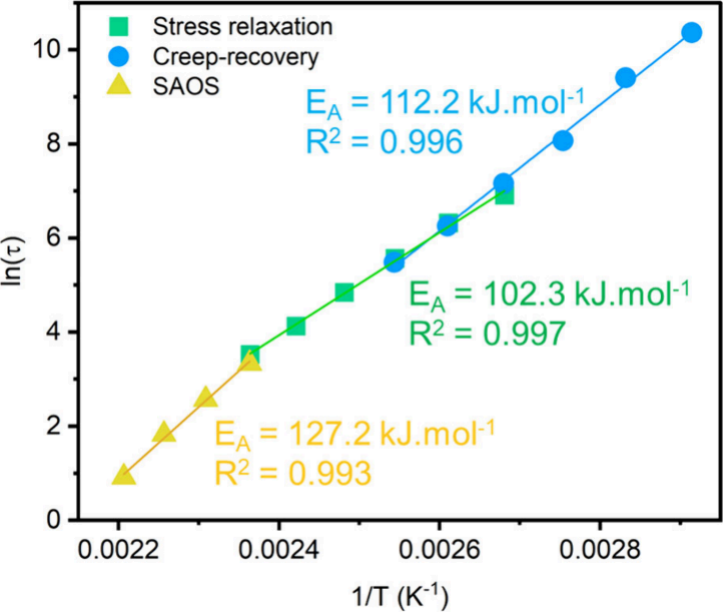
Plot of ln­(τ) as a function of temperature inverse (K^–1^) extracted from isothermal creep-recovery, stress relaxation and small amplitude oscillatory shear experiments at various temperature (from 80 to 180 °C) for CF_3_-*N,S*-acetal CAN. Reproduced from ref 120. Copyright 2024 American Chemical Society.

While most of the studies employed the characteristic relaxation time (τ* or ω_G′=G″_) to assess the Arrhenian dependency, it is recommended to plot η_0_ as a function of 1/T, because η_0_ also includes the temperature-dependence of the storage modulus. The Arrhenian-type evolution of zero-shear viscosity (ln­(η_0_)) law is verified in any case for vitrimers but only in a certain temperature range for dissociative CANs.^188^ Indeed, as the cross-link density depends on the ratio of association and dissociation rate constants (*k*_ass_, *k*_diss_), G′ can be only considered as a constant when *k*_ass_/*k*_diss_ ≫ 1. It has been shown that for dissociative CANs based on Diels–Alder exchange reactions,^189–191^ trans-*N*-alkylation,^192^ urea dissociation,^193^ or thiol-succinic anhydride exchange,^183^ an Arrhenian-behavior was observed over a certain temperature range. Nevertheless, at elevated temperature, the equilibrium is shifted toward the dissociated state (*k*_ass_/*k*_diss_ ≪ 1) and an abrupt drop of viscosity was recorded by DMA^188,194^ This equilibrium shift toward dissociated species was also detected for our trifluoromethylated *N,S*-acetal system in both DMA and SAOS experiments at elevated temperature.

It is noteworthy to mention that the flow activation energy is related to but does not exactly represent the activation energy (determined by molecular studies) of the chemical process itself.^195^ E_A_ values are dependent on a multitude of parameters such as the nature of the exchange,^32,169^ the cross-linking density of the network,^19^ the nature or absence/presence of catalyst,^78^ the stoichiometry of the exchangeable groups^34,36,50,166,196^ and the polymer matrices.^39^ This multiple factor dependency explains why, for an identical exchange reaction, activation energies can vary by several decades. *E*_a_ ranging from 25 to 160 kJ mol^–1^ have, for example, been reported for transesterification-based vitrimers.^32^

### CANs with Multiple Relaxation Modes

#### One Exchange Reaction with Different Rates/Mechanisms

The mechanistic pathway of an exchange reaction depends on multiple factors such as the presence of an activating neighboring group, the introduction of an exogenous catalyst, or the temperature. For instance, the covalent exchange in vinylogous urethane CANs can occur through two different transition states/intermediates. At low temperature, the exchange mechanism proceeds via the formation of an iminium whereas at high temperature the Michael exchange mechanism becomes predominant. Two activation energies can be measured depending on the predominant mechanism (Figure 22).^112,174^ This typical behavior highlights the importance of studying dynamic properties over a wide range of temperature.

**Figure 22:**
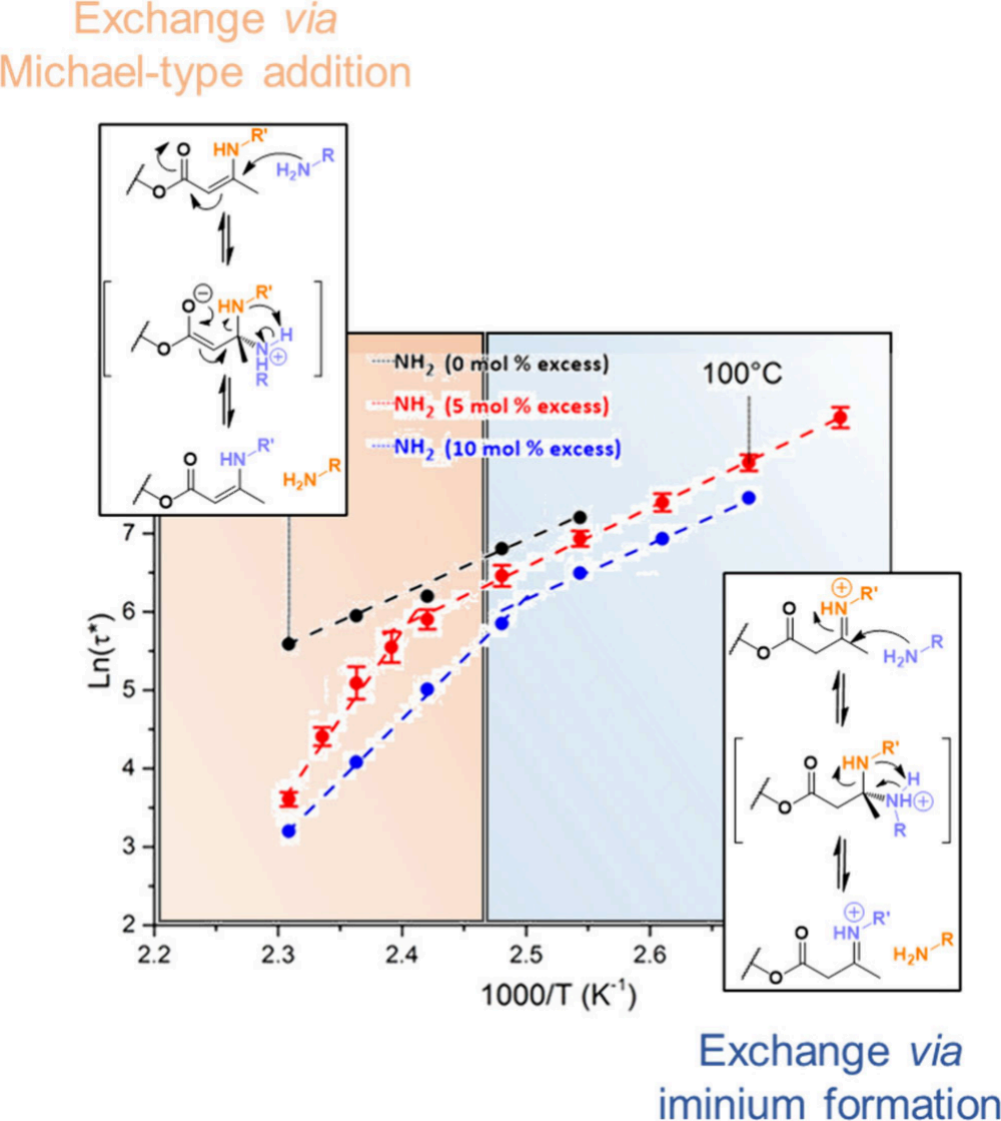
Non-Arrhenian behavior of a vinylogous urethane CAN comprising two distinct exchange mechanisms and kinetics depending on the temperature. Reproduced from ref 112. Copyright 2018 American Chemical Society.

Furthermore, Evans et al. investigated the relaxation of vitrimers with kinetically distinct boronic esters dynamic bonds.^197^ For CANs prepared by reacted α,ω OH-polydimethylsiloxane with either boric acid or three phenyl-substituted boronic acids (with different exchange kinetics) or a combination of boric acid with a phenyl-substituted one, the dynamic properties were almost identical to those of the CAN containing only the fastest exchangeable bonds. Following this work, the group of Du Prez studied in details the rheological behavior of a CAN incorporating both slow and fast exchanging moieties.^198^ This CAN was based on the dissociative transesterification of phthalate monoesters with β-amino alcohol (fast) and nonactivated (slow) alcohols. The authors mentioned that by using a general Maxwell model with two components, the two modes of response could be separately evaluated (eq 10).

10 $$G\left(t\right)=G_{0,fast}e^{-t/\tau_{fast}}+G_{0,slow}e^{-t/\tau_{slow}}$$

where τ_fast_ and τ_slow_ correspond respectively to the relaxation time of the dissociative and associative transesterification-based CAN. The different behavior observed in those two examples can be explained by the ratio employed between the fast and the slow exchangeable functions. With the transesterification-based CANs, only 20 mol % of rapid/dissociative moieties were used to build up the networks while PDMS-based CAN were prepared using a 1:1 molar ratio between both rapid/slow cross-linkers. Using the same exchange reactions and cross-linkers but on a different polymer backbone (poly­(*n*-butyl acrylate)^199^), the same research group was able to obtain two distinct relaxation modes even in equimolar conditions. To explain such results, the authors hypothesize that the presence of multiple dynamic linkages along a polymeric backbone and the ability of dynamic bonds to randomize at each cross-link site were critical to generate and control multimodal relaxations within in a single polymer network.

#### Masked Reactive Functions

One of the major challenges in the field of CANs is to finely trigger the covalent exchange processes, in other words, to start the material flow but solely when desired. In order to inhibit exchange at service temperatures and to directly access low viscosity at a specific temperature, blocking the exchanges has been considered. Recently, the team of F. Du Prez developed a CAN for which the exchangeable groups were released only at high temperature.^200,201^ In these vitrimers based on the exchange of vinylogous urethane moieties, no primary amine was initially present in the network. The formation of an imide function via intracyclization of two spatially closed amides led to the release of a free primary amine able to initiate the covalent exchange process. As the formation of the imide only occurs at high temperatures. The CAN prepared from this peculiar, masked group strategy exhibits globally a non-Arrhenian behavior but, above the amine release temperature, τ* follows an Arrhenius law. Alternatively, primary amine can be reversibly masked by the formation of ammonium acid–base complex using Brønsted acid such as benzoic or sulfonic acid derivatives.^202^

#### CANs with Multiple Exchange Reactions

The coupling of multiple exchange reactions in a unique CAN has been investigated to design materials with sufficiently fast relaxation so that they can be (re)­shaped using techniques commonly utilized with thermoplastics, such as extrusion or thermoforming.^203–209^ Indeed, the combination of two exchange reactions has been demonstrated to accelerate the relaxation of the material, probably due to simultaneous exchanges. However, the existence of several exchange reactions in these CANs renders the analysis of their dynamic behavior more complicated. To the best of our knowledge, these CANs have so far been evaluated using only the approach used for CANs involving only one type of exchange reaction. However, it could be interesting to develop similar strategies to the one described for systems with one exchange reactions and two distinct mechanisms or dynamic time scales to specifically evaluate this emerging multidynamic type of CANs.

### Evaluation of Dynamic Properties by Temperature Translation Factors

Using the time–temperature superposition (TTS) principle, we can evaluate rheological data in another way. Rheological analyses can be translated in time using a shift factor: a_T,Network_ and a_T_ for experiments performed in oscillatory (SAOS) and static (stress relaxation and creep/recovery) modes, respectively. In the case of dissociative CANs for which the storage modulus decreases with an increase in temperature, additional translation factors for modulus (b_T,Network_ and b_T_) have also been implemented. The TTS principle allows the construction of master curves over a wide range of frequencies. According to these definitions and considering a reference temperature T_0_ (generally the *T*_g_ in Williams–Landel–Ferry model^63,169^ ), G*­(ω, T_0_) and G­(t, T_0_) are determined using eqs 11 and 12.

11 $$G^{*}\left(\omega ,T_{0}\right)=\frac{G^{*}\left(\omega a_{T,Network},T\right)}{b_{T,Network}}$$

12 $$G\left(t,T_{0}\right)=\frac{G\left(\frac{t}{a_{T}},T\right)}{b_{T}}$$

In the case of associative CANs, b_T,Network_ and b_T_ theoretically have a value of 1 as G*­(ω) or G_0_ (t) is independent of temperature (in the rubbery region). The only parameters of interest are thus a_T,Network_ and a_T_ which represent the variation of relaxation time with temperature.^210^ Moreover, as observed for τ* and as expected for a parameter linked to relaxation caused by covalent exchanges, time shift factors should also follow the Arrhenius law (Figure 23). Viscous flow activation energy can thus be determined from the evolution of the time shift factors with 1/T.^20,211^

**Figure 23:**
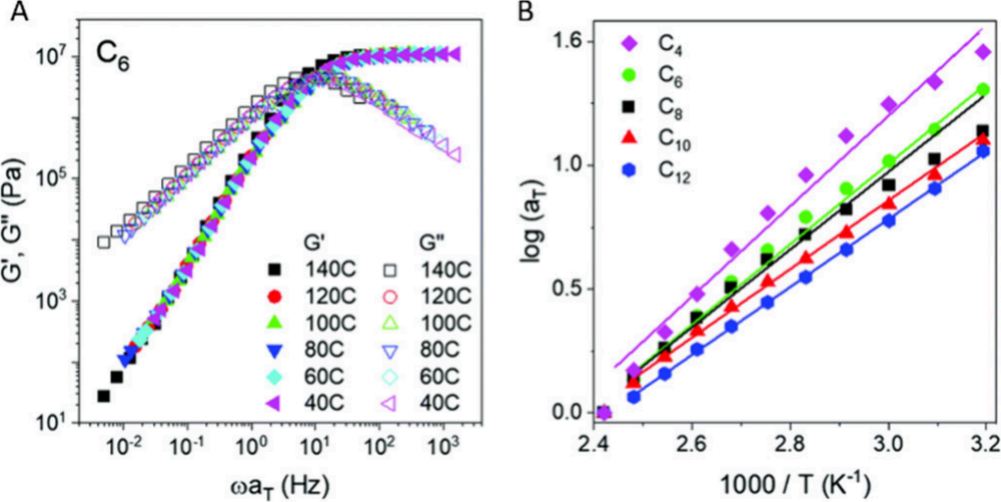
(A) Master curves of ethylene vitrimers based on exchangeable dioxaborolane cross-links. (B) Arrhenius plot of the shift factor a_T,Network_ as a function of temperature. Reproduced with permission from ref 211. Copyright 2021 Royal Society of Chemistry.

In the case of dissociative CANs, the variation of the modulus shift factors has to be taken into account, as this variation with temperature also impacts the determination of the zero-shear viscosity (η_0_). Montarnal et al. found out that, in a specific temperature range (before the terminal relaxation), b_T,Network_ and b_T_ also follow Arrhenius law. This was attributed to the fact that the cross-link density varies as the association constant of the reaction, which also obeys Arrhenius law.^169^ Since η_0_ depends on relaxation time and storage modulus values, the flow activation energy of the material corresponds to the sum of the activation energy determined from the temperature dependence of the relaxation time (obtained from the evolution of a_T,Network_ or a_T_) and of the activation energy determined from the shift of the storage modulus (determined from the evolution of b_T,Network_ or b_T_).^161^

### Activation Energy Discussion

Due to the evolution of viscosity of CANs with temperature, a high activation energy (E_A_) implies that a small increase in temperature induces a large drop in viscosity. Thus, CANs with very high E_A_ will show a significant difference in viscosity between the reshaping temperature and the service temperature.^212^ Controlling the activation energy thus appears as a desirable goal to obtain structurally stable CAN with limited creep below 100 °C and fast relaxation at processing temperature.

In addition to this preliminary remark, some trends emerged and thus can be applied to enable the design of high E_A_ CANs. For example, for similar polymer matrices based on the same exchange reaction, a higher cross-link density is associated with an increase in activation energy.^19,213^ Nevertheless when the polymer matrix is different, the E_A_ can be significantly modified even if the exchange mechanism remains identical. In the case of transamination, activation energies ranging from 68 kJ mol^–1^ for aliphatic vitrimers to 117 kJ mol^–1^ for PDMS-based vitrimers have been reported.^39^ For imine-based vitrimers, a variation of 30 kJ mol^–1^ was observed between materials based on slightly different amines. Furthermore, E_A_ is dependent on the exchange mechanism of the same reaction. For example, in the case of transamination of vinylogous urethanes, the protic mechanism (which proceeds via an iminium intermediate) is associated with an E_A_ of 60–70 kJ mol^–1^ whereas the aprotic mechanism (Michael-type addition) is associated with an E_A_ of 130–170 kJ mol^–1^.^112^ The type of catalysts used to promote exchanges was also shown to influence E_A_; although the catalyst concentration did not have much impact.^74,78^ These observations confirm that the exchange mechanism is a key element in the value of E_A_. Bates and co-workers showed that the p*K*_a_ of the Brønsted acid employed as catalysts in transesterification-based vitrimers strongly influenced both the activation energy and the relaxation times.^76^ Depending on the acid used and its corresponding p*K*_a_, a relationship of proportionality has been demonstrated between the E_A_ and the strength of the acid. Indeed, acid with higher p*K*_a_ exhibited lower E_A_ and longer relaxation times, while the opposite trend was observed with stronger acids. However, according to the authors this relationship may just be fortuitous in this study and should be confirmed on other systems.

The above-mentioned results and trends are taken from articles that did not specifically focus on controlling the variation of E_A_. This explains why predicting the E_A_ value of a CAN is still difficult. A systematic analysis of each parameter influencing the activation energy, in spite of its complexity of implementation, is necessary if one hopes to achieve a precise understanding of the variation of E_A_ and the means to design CANs with desired E_A_ values. Several research groups have focused their attention on modeling CAN dynamic behavior.

Based on the observation that E_A_ is dependent on many factors, Du Prez et al. suggested that the Arrhenius theory may be oversimplified.^198^ Hence as previously done both by Bowman et al. for Diels–Alder CANs^214^ and by Tibitt and co-workers for boronic esters CANs,^215^ the evolution of τ* with temperature was analyzed using the adjusted Eyring eq (eq 13) considering that the rate constant of the reaction was equal to 1/τ*.

13 $$\tau^{*}=\frac{\hbar }{\kappa K_{b}T}e^{-\Delta S^{‡}/R}e^{\Delta H^{‡}/RT}$$

with ΔS^‡^ and ΔH^‡^ being the entropy and enthalpy of reaction, respectively, K_b_ being the Boltzmann constant (1.38 × 10^–23^ J·K^–1^), ℏ being the Planck constant (6.626 × 10^–34^ J·s) and κ being the transmission coefficient.

The determination of ΔH^‡^ and ΔS^‡^ by the plotting of ln­(τ*) as a function of 1/T allows us to discuss more precisely the influence of NGP, catalyst, cross-linking, or polymer structure effect on the exchange reaction. Indeed, the variation of ΔH^‡^ can be attributed to higher or lower activation barrier whereas the variation of ΔS^‡^ can be linked to structural variation. The sign of ΔS^‡^ can also give an indication of the type of the mechanism. A negative ΔS^‡^ is expected for a dissociative mechanism, while an associative mechanism should be characterized by a positive entropy of reaction. Nevertheless, one should keep in mind that these interpretations are based on the assumption that the rate constant of the exchange reaction is directly proportional to the relaxation time. However, as previously discussed, the relaxation time is dependent on multiple parameters and such an interpretation should be considered with care. In addition to this study mainly based on the direct analysis of rheological results, researchers have attempted to associate the relaxation process observed in CANs to theoretical polymer models. This kind of work is out of the scope of this review but could provide key elements to achieve control over dynamic properties. The authors invite the readers to refer to the following references for insights into the theoretical modeling of thermal CAN behaviors.^63,161,163,210,211,216,217^

## Typical Study of a CAN: A Guideline

Following the presentation of the different analyses that can be performed to study CAN dynamic properties and their physical behavior with temperature, it is possible to establish a general guideline to accurately characterize a CAN (Figure 24). Prior to material synthesis and characterization, it is recommended that one characterize the feasibility of the exchange reactions at the molecular level. Thermodynamic and kinetic parameters as well as the influence or necessity to use catalysts/neighboring groups are thus identified, allowing an optimized CAN design. Once a material is obtained, it is necessary to first demonstrate that a cross-linked chemical network has indeed been formed by running swelling tests and calculate both the swelling index and the gel content. Structural characterization techniques, although limited in number (FTIR, RAMAN, HR-MAS NMR), should also be used to evaluate the extent of reaction and the occurrence of potential side reactions. TGA and DSC analyses can be then employed to determine the main transitions (*T*_g_, *T*_m_) and the suitable temperature window for rheological characterizations (linear rheology experiments). DMA and mechanical tests should be then performed to evaluate material properties over a wide range of temperatures and deformations. In a second step, to test the efficiency of the dynamic exchange in the network, reshaping and reprocessing tests can be carried out. A simple visual check will give an idea of whether the material can be reprocessed under these conditions (based on the literature or small molecule studies) or not. Generally, relaxation occurs on a time scale comparable to that of reshaping, although complete relaxation of the network is not necessary for effective reshaping. The order of magnitude of the relaxation times, once estimated, can be used to determine the most suitable rheological analysis to characterize the flow of the material and the temperature dependency. In the case of associative CANs or vitrimers, an Arrhenian-type behavior (linear evolution of ln­(η0) as a function of inverse temperature) must be obeyed, whereas a nonlinear evolution of the viscosity can be observed in the case of dissociative CANs. The nature and thermodynamic parameters of the postulated mechanism can then be compared with molecular investigations and DFT studies and the rheological observations (evolution of G_0(_(t) or G′(ω) with temperature) to conclude precisely on the associative or dissociative character of the CAN. The comparison of FTIR spectra, gel content and swelling index, TGA and DSC thermograms as well as DMA (and especially the value of storage modulus on the rubbery plateau), mechanical tests and rheological analyses carried out on the initial material and on the reshaped materials will allow conclusions to be drawn on the efficiency of the reprocessing and on the ″viability″ of the recycled material.

**Figure 24:**
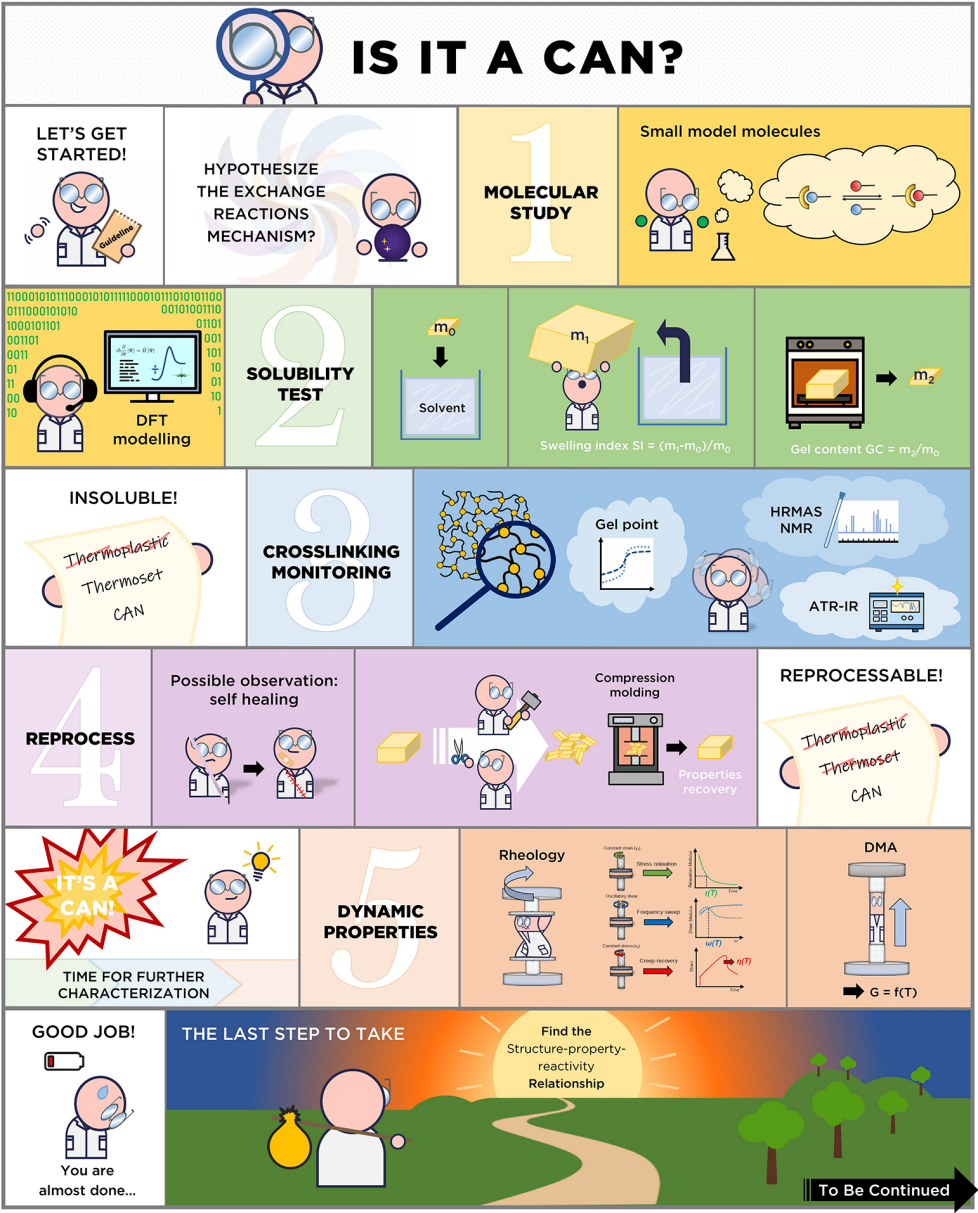
“How to characterize Covalent Adaptable Networks” comic strip.

## Conclusion

The present tutorial review provides a *vade mecum* to the neophytes in the field who would like to start working on dynamic networks based on covalent bonds (CANs). After a brief description of the general properties of thermoplastics and thermosets, characterization methods and models used to describe CAN rheological behavior are presented. Stress relaxation, creep/recovery, and small amplitude oscillatory shear analyses are the most common rheological measurements used to characterize the terminal relaxation of CANs. These techniques enable us to monitor the evolution of viscosity as a function of temperature (or time). Depending on the selected experiments, relaxation time (stress relaxation), deformation rate (creep recovery), or crossover frequency (SAOS) is determined. Even if one of these parameters is sufficient to determine material thermal behaviors as they are interrelated, each experiment has a more favorable time scale. Complementary analyses such as DMA, temperature-controlled FTIR spectroscopy, or broadband dielectric spectroscopy (BDS) have also recently been used to characterize the dynamic behavior of CANs and to associate the rheological behavior to chemical exchange processes. Following the description of suitable methodologies and models, we proposed guidelines to characterize CANs properly and rapidly. This user guide starts with the monitoring of the network formation and the demonstration of the insolubility of the CANs and goes to the investigation of the exchange mechanism. Despite their promising properties and the ambition to replace classical nonrecyclable thermosets, CANs are still “research objects” with only limited industrial applications. Several factors can explain the reluctance of materials manufacturers and end-users to adopt CANs. The (re)­processing of CANs is generally too slow (generally from a few minutes to hours) and is not adapted to thermoplastic processing technologies (extrusion and injection). CANs do not match the thermo-mechanical properties of high-performance thermosets due to creep, and a trade-off between stability (thermal and mechanical) and recyclability is often needed. There is also a lack of knowledge of the dynamic properties and aging (fatigue, number of cycle possible, catalyst deactivation or leaching, etc.) of such materials. Hence, the control of the dynamic properties of CANs and more specifically of the relaxation time and flow activation energy and of their creep and fatigue behavior is a key research field. This review gives guidelines to standardize the way of characterizing CANs and the authors hope that it will allow material comparisons and facilitate the understanding of properties evolution. Studies in which CAN parameters (exchange reaction, cross-link density, exchangeable function concentration, effect of multiple exchange reactions, nature of the mechanism, neighboring group participation) are individually evaluated should enable to promote the industrial development of CANs.
